# Defined culture conditions robustly maintain human stem cell pluripotency, highlighting a role for Ca^2+^ signaling

**DOI:** 10.1038/s42003-025-07658-z

**Published:** 2025-02-18

**Authors:** Ilse Eidhof, Benjamin Ulfenborg, Malin Kele, Mansoureh Shahsavani, Dania Winn, Per Uhlén, Anna Falk

**Affiliations:** 1 Department of Neuroscience, Karolinska Institutet, Stockholm, Sweden; 2 Department of Medical Biochemistry and Biophysics, Karolinska Institutet, Stockholm, Sweden; 3 School of Bioscience, University of Skövde, Skövde, Sweden; 4 Department of Experimental Medical Science, Lund University, Lund, Sweden; 5 Present Address: BioLamina AB, Sundbyberg, Sweden; 6 Present Address: Department of Molecular Medicine and Surgery and Center for Molecular Medicine, Karolinska Institutet, Stockholm, Sweden

**Keywords:** Pluripotent stem cells, Research management, Stem-cell research

## Abstract

Induced pluripotent stem cells (iPSCs) have significant potential for disease modeling and cell therapies. However, their wide-spread application has faced challenges, including batch-to-batch variabilities, and notable distinctions when compared to embryonic stem cells (ESCs). Some of these disparities can stem from using undefined culture conditions and the reprogramming procedure, however, the precise mechanisms remain understudied. Here, we compared gene expression data from over 100 iPSC and ESC lines cultivated under undefined and defined conditions. Defined conditions significantly reduced inter-PSC line variability, irrespective of PSC cell type, highlighting the importance of standardization to minimize PSC biases. This variability is concurrent with decreased somatic cell marker and germ layer differentiation gene expression and increased Ca^2+^-binding protein expression. Moreover, SERCA pump inhibition highlighted an important role for intracellular Ca^2+^ activity in maintaining pluripotency gene expression under defined conditions. Further understanding of these processes can help standardize and improve defined hPSC culture conditions.

## Abstract (web summary)

Comparative analysis of 100+ PSC lines highlights biological differences between defined and undefined culture conditions. Standardization reduces PSC variability, maintains self-renewal, and enhances reproducibility with a role for Ca2+ signaling.

## Introduction

The reprogramming of adult skin cells to obtain induced pluripotent stem cells (iPSCs), which earned the Nobel Prize in 2012, has opened a plethora of possibilities^1^. This remarkable achievement brought us significantly closer to cell transplantation therapies and provided deeper insight into the molecular and cellular mechanisms underlying human diseases and early brain development. However, the lack of standardization and difficulties in maintaining human iPSC pluripotency and self-renewal under undefined culture conditions have restricted the clinical applicability of iPSCs since their discovery. The use of fibroblast feeder cells, Matrigel, and undefined media containing fetal bovine serum (FBS) negatively affected the reproducibility of iPSC lines^2,3^. These can lead to high batch-to-batch variability in the differentiation potential of iPSC lines between different research laboratories, increase the risk of pathogen contamination, and induce immunogenicity^2,3^. The high culture costs associated with undefined iPSC matrices and media further limit their scalability^2,3^. To address these challenges, recent efforts have been made by large networks, including the International Society for Stem Cell Research (ISSCR) (https://www.isscr.org/standards-document) and the European College of Neuropsychopharmacology Network to provide researchers with best practices and recommendations for the use of human stem cells in research^4^. Moreover, an increasing amount of researchers, including ourselves, started to utilize xeno-free, fully defined (FD) iPSC culture matrices, such as laminin 521 (LN-521) and vitronectin, and FD iPSC culture media, such as essential 8 (E8), to facilitate the robust generation and maintenance of iPSCs^2,3,5^. Despite these advancements, FD culture conditions are not universally adopted, and their long-term suitability, scalability, and the underlying biological processes for maintaining homogenous iPSCs from diverse origins, remain poorly understood. Additionally, growing evidence suggests that iPSCs and Embryonic Stem Cells (ESCs) harbor significant epigenetic and functional distinctions, including somatic cell memory retention^6,7^. This poses potential implications for the function, differentiation potential, and broader application of iPSCs. While it is believed that some of these disparities arise during the reprogramming procedure, the precise mechanisms behind these differences remain understudied. Gaining a deeper understanding of these processes will be crucial in the standardization and improvement of iPSC culture conditions. This will ultimately help to enhance the rigor, reproducibility, accuracy, and unambiguous reporting of iPSC research, and support the clinical applications of iPSCs.

Here, we performed a comparative analysis of gene expression data obtained from over 100 human iPSC and ESC lines cultivated in undefined and/or FD conditions, that have been collected over the course of a decade. We used these datasets to characterize biological differences between PSCs cultured in (un)defined conditions, and the processes required to maintain PSC pluripotency and self-renewal capacity. Our research sheds light on the importance of standardization to minimize biases and variations between PSC lines that are not caused by differences in genetic background.

## Results

### Defined culture conditions promote greater uniformity among PSC lines

**Fig. 1:**
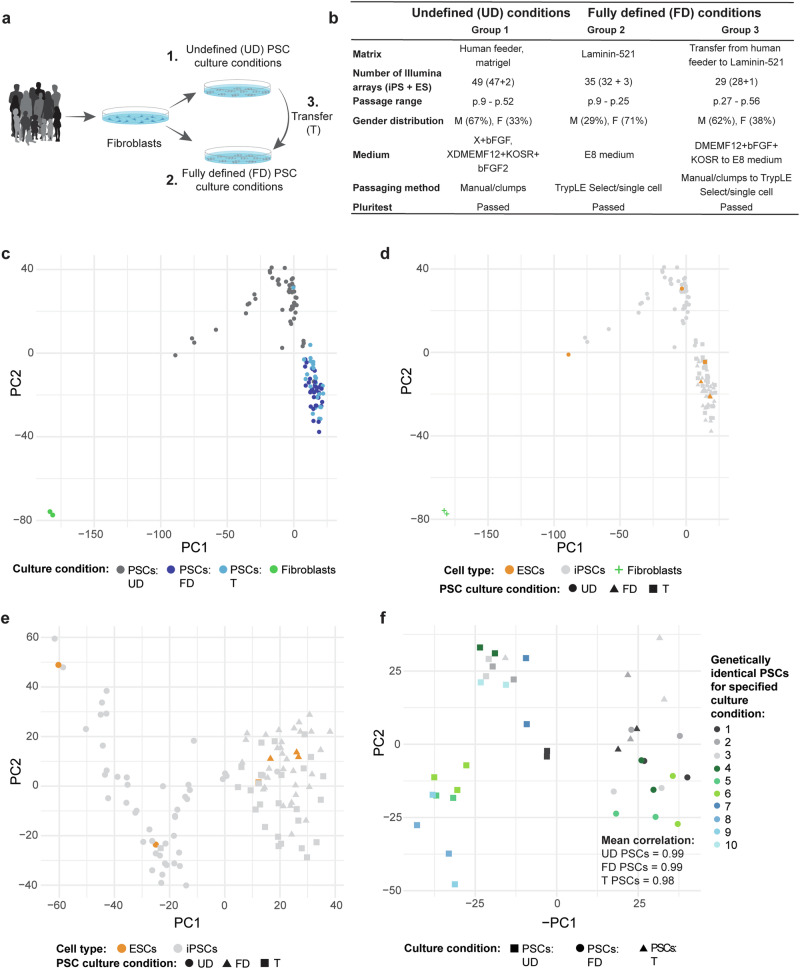
Defined culture conditions promote greater uniformity among PSC lines compared to undefined culture conditions. **a** Schematic representation of the three groups included in the analysis. **b** Table summarizing the exact culture conditions and number of PSC lines for each group. **c** PCA visualization of PSC lines derived and cultured in 1. Undefined culture conditions (in gray), 2. Defined culture conditions (in dark blue), 3. Transferred from undefined to defined culture conditions (in light blue) and 4. Fibroblasts (in green). **d** PCA visualization as in C, with ESCs (in orange) and iPSCs (in gray) highlighted. **e** PCA visualization as in D of ESC (in orange) and iPSC (in gray) lines derived and cultured in 1. UD culture conditions (in circles), 2. FD culture conditions (in triangles) and 3. Transferred from UD to FD culture conditions (in squares). Fibroblast samples were excluded from the visualization (**f**). PCA visualization of genetically identical PSC lines within dataset derived and cultured in 1. Undefined culture conditions (in squares), 2. Defined culture conditions (in circles) and 3. Transferred from undefined to defined culture conditions (in triangles). The color codes represent different technical or biological replicates. When there is a similar color code in one of the three groups (UD, FD, or T), the data originates from either different iPSC clones of the same individual or iPSCs from monozygotic twins. Of note, there is no overlap in biological replicate samples between the three culture condition groups.

To investigate pathways involved in maintaining PSC pluripotency and self-renewal in defined culture conditions, we utilized our substantial collection of Illumina gene expression array data obtained from over 100 healthy human control iPSC, neurogenetic patient iPSC, and ESC lines. From 16 of the iPSC lines, our dataset contained gene expression data of 1–2 additional clones, resulting in a total of 36 unique individuals represented in our study. The PSC lines were cultivated under undefined (UD), FD, or transferred (T) from UD to FD culture conditions (Fig. 1a, b). All PSC lines were cultured in the presence of bFGF. The iPSCs showed a morphology resembling human ESCs, with large nuclei, compact cytoplasm, and sharp edges. All iPSCs/ESCs included in this study met the PluriTest criteria for pluripotency, as evidenced by their Pluripotency and Novelty scores, PSC marker expression, and normal karyotypes (Supplementary Data 1). A comprehensive overview of the samples and culture conditions is presented in Fig. 1b, with detailed information in Supplementary Data 1.

To identify factors contributing to PSC line variability, we performed principal component analysis (PCA) on the Illumina array dataset. In this analysis, we incorporated Illumina array data from two human skin fibroblast cell lines. The PCA results revealed that the primary source of variability (Principal Component 1 (PC1), 20%) between the different samples was determined by cell type and PSC culture conditions (Fig. 1c). In line with meeting the PluriTest criteria, PSCs formed a distinct cluster, clearly separated from the skin fibroblast cell lines. PC1 also separated a more widespread UD PSC cluster from a more homogenous FD and T PSC cluster (Fig. 1c, in gray), with UD PSCs positioned slightly closer to fibroblast cells. The second most influential factor in PCA (PC2, accounting for 13% of the variability), was associated with cell culture conditions. PC2 delineated the UD PSC group (in gray) from the FD and T PSC groups (Fig. 1c, dark/light blue), suggesting that these groups can be characterized by overall differences in gene expression. Interestingly, the FD PSC group (dark blue) and T PSC group (light blue) overlapped substantially, suggesting that these two conditions are very comparable (Fig. 1c). To confirm the differences between the skin fibroblast samples and the PSC sample groups, we examined the expression of 10 well-known skin fibroblast marker genes (*VIM*, *PDGFRA*, *PDGFRB*, *ANXA2*, *FAP*, *COL1A1*, *ELN*, *S100A4*, *ACTA2* and *LAMB1*). All genes were significantly and strikingly downregulated in all PSC sample groups compared to fibroblasts (Supplementary Data 2). Most markers, including *VIM*, *PDGFRA*, *COL1A1*, *ACTA2* and *LAMB1* were also significantly downregulated in FD compared to UD PSC culture conditions (Supplementary Fig. 1). None of the genes were differentially expressed between FD and T PSC culture conditions. These findings collectively demonstrate that UD and FD PSCs exhibit distinguishable characteristics, with UD PSCs displaying slightly elevated expression of somatic cell markers. Noteworthy, this elevated somatic cell marker expression did not reach the fibroblast cell line levels.

We pondered whether iPSCs in UD culture conditions exhibited a slightly higher propensity for retaining somatic cell memory compared to iPSCs in FD culture conditions. To address this question, we investigated divergences between iPSCs and ESCs cultured in FD and UD conditions in PCA (Fig. 1d–e). The analysis reaffirmed the general clustering of PSCs, away from skin fibroblasts (Fig. 1d). Similar to UD iPSCs, UD ESCs displayed a more widespread distribution compared to their more uniform FD/T counterparts (Fig. 1d, e). Moreover, all groups showed a high mean correlation among individual samples, with mean correlations of 0.98 among UD iPSCs, 0.95 among UD ESCs, 0.98 among FD iPSCs, and 0.98 among FD ESCs, respectively. This implies high similarity among individual samples within these groups. To investigate whether iPSCs and ESCs cultured in either UD or FD conditions were molecularly distinct, we continued conducting differential gene expression (DGE) analysis. 57 Differentially Expressed Genes (DEGs) were identified between UD iPSCs and UD ESCs (Supplementary Data 2). Notably, no DEGs were identified between FD iPSCs and FD ESCs, affirming that these two groups have a high molecular resemblance. We proceeded to investigate whether the 57 DEGs between iPSCs and ESCs in UD culture conditions were enriched for known skin-fibroblast marker genes. The analysis affirmed that this was not the case. Instead, Kyoto Encyclopedia of Genes and Genomes (KEGG) pathway database analysis revealed an enrichment for pancreatic cancer (KEGG:05212, *p* = 0.007) and bladder cancer (KEGG:05219, *p* = 0.027). These findings collectively suggest that UD culture conditions augment variability between iPSCs and ESCs and elevate somatic cell marker expression compared to FD culture conditions. FD culture conditions reduced variability, yielding a homogenous pool of iPSCs and ESCs with uniformly low somatic marker expression.

As culture conditions emerged as major factor influencing PSC variability, with only minor differences between UD ESCs and iPSCs, we pooled all PSC samples. Our focus shifted then specifically to PSC lines in PCA, for a more thorough analysis of potential confounding factors contributing to PSC sample variation (Fig. 1e). This revealed once again that the primary source of variability, as explained by PC1 (17%), could be attributed to transcriptional differences between PSCs cultivated under FD and UD conditions (Fig. 1e, Supplementary Fig. 2A). Other confounding variables, such as diagnosis, donor sex, passage number, and/or PSC line origin, had minimal impact (Supplementary Fig. 2B–E). Consistently, PSCs cultured in FD conditions (dark blue) or those transferred to FD culture conditions (light blue) exhibited higher homogeneity and tighter clustering than UD PSCs (gray) (Supplementary Fig. 2A). Thus, despite meeting pluripotency quality criteria, UD PSCs displayed considerably larger variability compared to FD PSCs.

To further investigate the greater heterogeneity observed in samples from UD culture conditions, we examined whether genetically identical samples exhibited closer clustering and higher correlation in FD culture conditions compared to UD conditions (Fig. 1f). These genetically identical PSC lines included iPSCs derived from monozygotic twins and different iPSC clones originating from the same donor. Notably, none of the genetically identical PSC sample duplicates were shared between the FD and UD culture conditions. Genetically identical PSC lines demonstrated close clustering in PCA, with a mean correlation of 0.99 both for PSCs cultured under FD and UD conditions and a mean correlation of 0.98 for PSCs transferred from UD to FD conditions (Fig. 1f). This suggests that genetically identical PSC samples show consistent gene expression profiles, regardless of their exact culture conditions. Thus, the greater heterogeneity observed among PSCs under UD culture conditions are likely due to a higher overall inter-PSC line variability.

In conclusion, FD culture conditions promoted homogeneity between PSC lines.

### Defined culture conditions show reduced expression of signaling pathways that regulate germ-layer differentiation

**Fig. 2:**
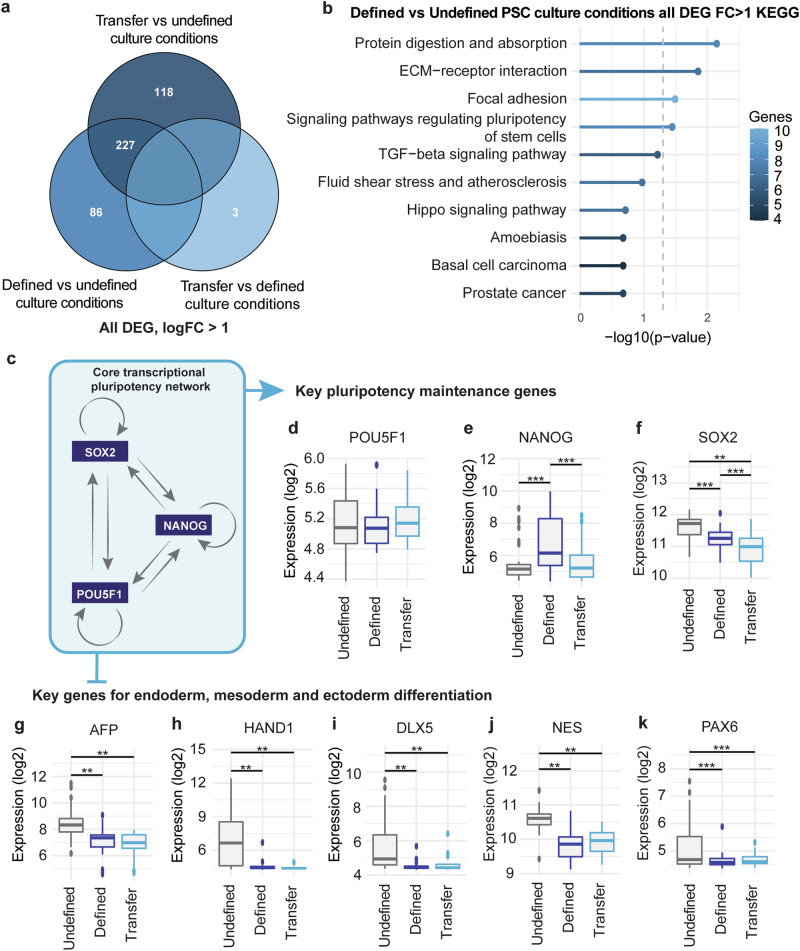
FD culture conditions show reduced expression of signaling pathways that regulate germ-layer differentiation. **a** VENN diagram showing the total number of DEGs passing a log foldchange >1 between the indicated PSC culture conditions. **b** KEGG pathway analysis on DEGs between PSC established in FD and UD culture conditions. **c** Schematic overview of the core transcriptional network regulating stem cell pluripotency. Log2 microarray expression values of *POU5F1* (**d**), *NANOG* (**e**), and *SOX2* (**f**) that make up the core network in (**c**). for the indicated PSC culture conditions. Log2 microarray expression values of the endodermal marker *AFP* (**g**), mesodermal markers *HAND1* (**h**) and *DLX5* (**i**), and ectodermal markers *NES* (**j**) and *PAX6* (**k**) for the indicated PSC culture conditions. **d–k** Statical testing: one-way ANOVA followed by Tukey HSD for post-hoc testing: **p < 0.01, ***p < 0.001. Boxplots: central line is median, the box limits are the lower and upper quartiles, the whistlers represent 1.5 times the IQR, and points are samples further away.

We next performed DEG analyses to identify biological processes contributing to increased inter-PSC line homogeneity under FD conditions. A total of 313 DEGs (fold change > 1, *p* < 0.05) were identified between the UD and FD PSC groups (Fig. 2a, Supplementary Data 2), consisting of 172 downregulated and 141 upregulated genes (Supplementary Fig. 3A, B). 345 DEGs were identified between the UD and T PSC groups (Fig. 2a, Supplementary Data 2), including 221 downregulated and 124 upregulated genes (Supplementary Fig. 3A, B). Interestingly, only 3 genes were differentially expressed between FD and T PSCs (Fig. 2a, Supplementary Data 2), including two downregulated and one upregulated gene(s) (Supplementary Fig. 3A, B). This reaffirms a strong similarity between these two groups. Indeed, 227 of the 313 DEGs (72.5%) overlapped between FD and T PSCs (Fig. 2a). Among overlapping DEGs, 146 and 81 were up- and down-regulated, respectively (Supplementary Fig. 3A, B).

We continued asking whether the 313 DEGs between UD and FD PSCs were significantly enriched for KEGG pathways. Significant enrichments were found for: protein digestion and absorption (*p* < 0.01), ECM-receptor interaction (*p* < 0.05), focal adhesion (*p* < 0.05), signaling pathways regulating the pluripotency of stem cells (*p* < 0.05), and others (Fig. 2b). A similar analysis of 345 DEGs between T and UD PSCs revealed similar processes: focal adhesion (*p* < 0.01), signaling pathways regulating the pluripotency of stem cells (*p* < 0.01), p53 signaling pathway (*p* < 0.05), and protein digestion and absorption (*p* < 0.05) (Supplementary Fig. 3C).

These findings prompted us to examine genes that constitute the core transcriptional network responsible for maintaining pluripotency (Fig. 2c). While *POUF51* expression showed no significant changes (Fig. 2d), NANOG was upregulated (*p* < 0.001, Fig. 2e) in FD PSCs, whereas SOX2 was downregulated in both FD PSCs (*p* < 0.001) and T PSCs (*p* < 0.01, Fig. 2f). This indicates that culture conditions influence pluripotency gene expression.

To examine the downstream effects of DEGs in the pluripotency network, we analyzed common early lineage markers. Interestingly, a significant proportion of these, including the endodermal marker *AFP* (*p* < 0.01), mesodermal markers *HAND1* (*p* < 0.01) and *DLX5* (*p* < 0.01), and ectodermal markers *NES* (*p* < 0.01) and *PAX6* (*p* < 0.001), were significantly downregulated in FD and T PSCs (Fig. 2g–k). No significant changes were observed for endodermal markers *AMY1A*, *FOXA2*, and *PDX1*; mesodermal marker *FLT1*; and ectodermal markers *GFAP* and *SOX1* (Supplementary Fig. 3D–I).

These findings suggest that the larger variability in PSCs maintained under UD culture conditions may be attributed to the presence of a heterogenous population consisting of both PSCs and PSCs that have initiated or committed to differentiation.

### DEGs identified between defined and undefined PSC culture conditions are strongly interconnected at protein level and involved in shared biological processes

**Fig. 3:**
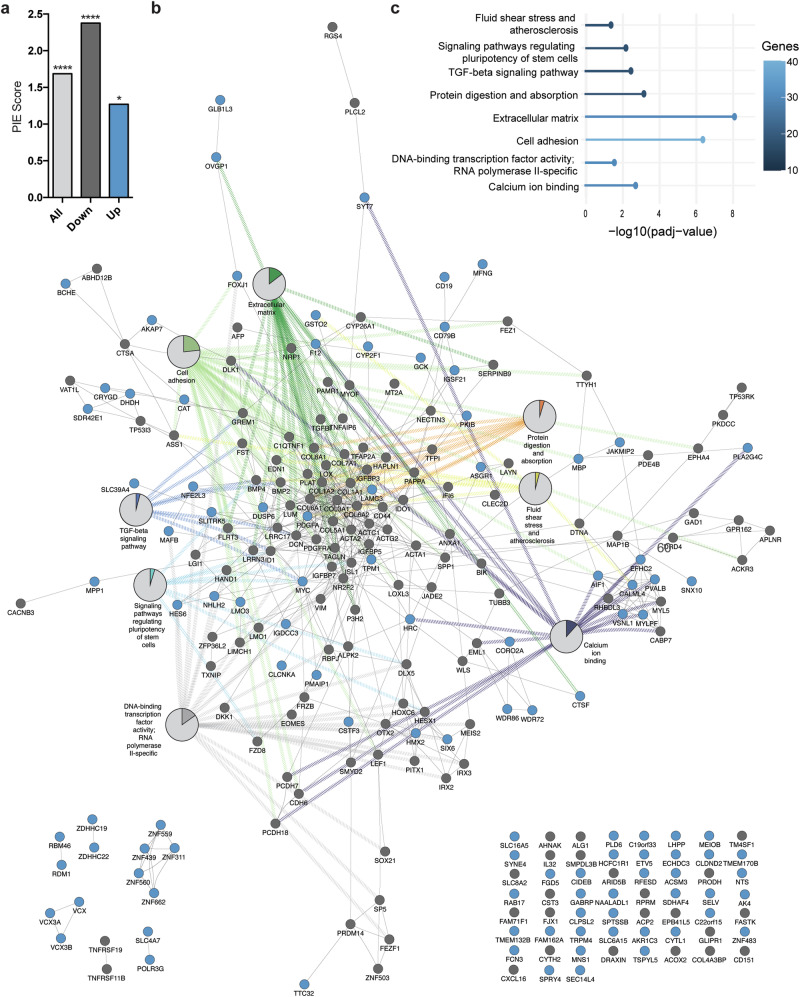
DEGs between FD and UD PSC culture conditions are strongly interconnected at protein level and function in shared biological processes. **a** Physical interaction enrichment (PIE) score of all (in light gray), downregulated (in dark gray), and upregulated (in blue) DEGs between FD and UD PSC culture conditions. *p < 0.05, ****p < 0.0001, based on 10,000 repetitions. **b** Interaction network of proteins differentially expressed between FD and UD PSC culture conditions and their significant associated GO terms. Pie charts display the percentage of proteins within the PPI network associated with the indicated GO term. **c** Significantly enriched GO terms for the large PPI network displayed in (**b**).

Understanding protein-protein interactions (PPIs), is crucial for elucidating mechanisms that maintain robust, homogenous PSC populations under FD culture conditions. To evaluate whether our list of DEGs in FD culture conditions showed significant molecular connectivity, we first collected PPIs using the Genemania plugin in Cytoscape^8^. Then, we explored which PPIs were more enriched among our list of DEGs compared to what would be expected by chance alone through a bootstrap analysis^9^. Significant PPIs were integrated into a reference network containing a total of 247 nodes and 645 edges (Fig. 3). These included: physical protein interactions (*p* < 0.0001), predicted protein interactions (*p* < 0.0001), shared protein domains (*p* < 0.0001), pathway interactions (*p* < 0.0001), and co-localized proteins (*p* < 0.0001) (Supplementary Fig. 4A). The resulting PPI network included a large module comprising 172 nodes and 618 edges, and six smaller modules comprising two or more nodes. A total of 59 proteins were not associated with any of the modules (Fig. 3b). The physical interaction enrichment (PIE) score^9^ was recalculated for the complete integrated PPI network. This yielded a PIE score of 1.7 (*p* < 0.0001), confirming significant molecular connectivity (Fig. 3a).

We next examined whether the identified PPI modules represent specific biological processes using Gene-Ontology (GO) analysis. The large protein module exhibited significant enrichment for processes such as signaling pathways regulating the pluripotency of stem cells (*p* < 0.01), TGF-beta signaling (*p* < 0.01), cell adhesion (*p* < 0.0001), extracellular matrix (*p* < 0.0001), DNA-binding transcription factor activity: RNA polymerase II-specific (*p* < 0.05), protein digestion and absorption (*p* < 0.001), fluid shear stress and atherosclerosis (*p* < 0.05) and calcium ion (Ca^2+^)-binding (*p* < 0.01). Interestingly, both upregulated (blue) and downregulated (dark gray) DEGs were represented within this network, indicating biological overlap (Fig. 3b, c). Nevertheless, the PIE score of downregulated genes markedly increased to 2.4 (*p* < 0.0001), whereas that of upregulated genes decreased to 1.3 (*p* < 0.05), compared to all DEGs (Fig. 3a). This trend was also observed in the PIE score analysis of distinct PPI categories (Supplementary Fig. 4B, C). Physical protein interactions (*p* < 0.0001), predicted protein interactions (*p* < 0.0001), shared protein domains (*p* < 0.0001), pathway interactions (*p* < 0.01), and colocalized proteins (*p* < 0.0001) were significantly enriched among the downregulated genes (Supplementary Fig. 4B). None of the unique PPI categories was significantly enriched among the upregulated genes under FD PSC culture conditions (Supplementary Fig. 4C).

**Fig. 4:**
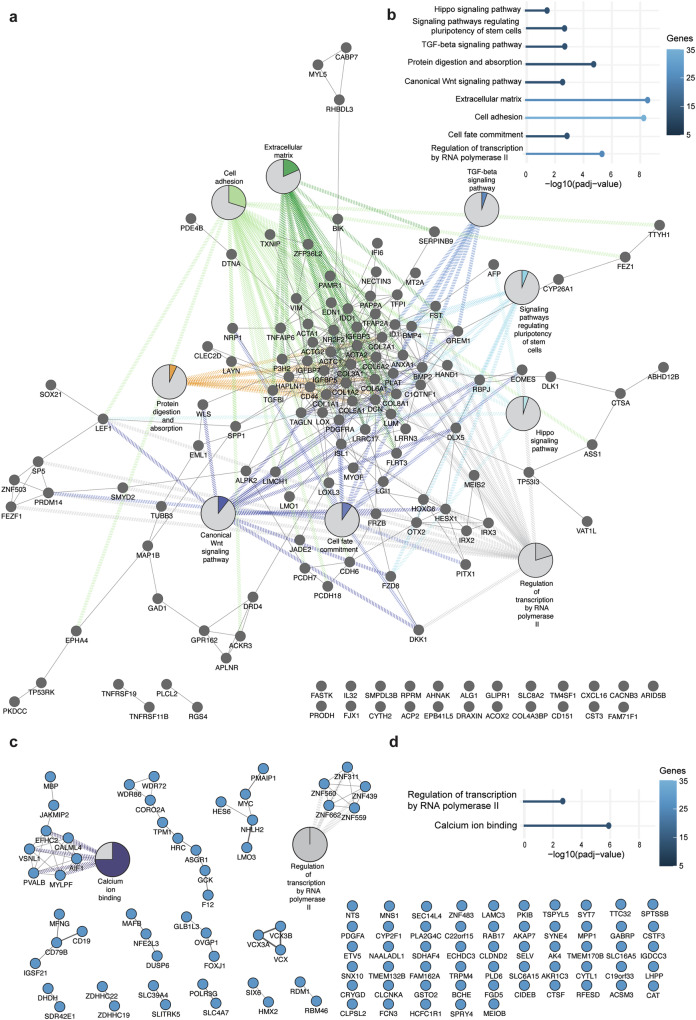
Up- and downregulated genes between FD and UD PSC culture conditions are strongly interconnected at protein level but are associated with distinct biological processes. **a**,** b** Analysis performed with downregulated genes in FD PSC culture conditions. **a** Interaction network of proteins downregulated in FD PSC culture conditions and their significant associated GO terms. Pie charts display the percentage of proteins within the PPI network associated with the indicated GO term. **b** Significantly enriched GO terms for the large PPI network displayed in (**a**). **c**, **d** Analysis performed with upregulated genes in FD PSC culture conditions. **c** Interaction network of proteins upregulated in FD PSC culture conditions and their significant associated GO terms. Pie charts display the percentage of proteins within the PPI network associated with the indicated GO term. **d** Significantly enriched GO terms for the PPI network displayed in (**c**).

Following these results, we investigated whether different biological processes at protein network level were specifically associated with down- and upregulated genes under FD PSC culture conditions. For this purpose, separate PPI networks were built for the down- and upregulated genes (Fig. 4). The downregulated genes formed a large and strongly interconnected PPI network, along with two small PPI modules, comprising 141 nodes and 414 edges (Fig. 4a). 23 nodes did not interact with any of the modules in the PPI network (Fig. 4a). GO and KEGG pathway analyses of the large PPI module revealed a striking overlap with significantly associated GO terms linked to the PPI network composed of all DEGs in Fig. 3b. Signaling pathways regulating the pluripotency of stem cells (p.adj < 0.01), TGF-beta signaling (p.adj < 0.01), protein digestion and absorption (p.adj < 0.0001), extracellular matrix (p.adj < 0.0001), cell adhesion (p.adj < 0.0001), and negative regulation of transcription by RNA polymerase II (p.adj < 0.0001) were all significantly associated with this PPI module (Fig. 4a, b). Furthermore, this PPI module was also significantly enriched with unique KEGG pathway terms linked to pluripotency and differentiation, such as the Hippo signaling pathway (p.adj < 0.05), canonical Wnt signaling pathway (p.adj < 0.01), and cell fate commitment (p.adj < 0.001) (Fig. 4a, b).

The upregulated genes under FD PSC culture conditions formed 14 small PPI modules with 106 nodes and 67 edges, while 55 nodes remained unconnected (Fig. 4c). GO and KEGG pathway analyses were performed for modules consisting of five or more nodes. The significantly associated GO terms overlapped with those associated with the PPI network consisting of all DEGs in Fig. 3b. One module consisting of five proteins was significantly enriched for transcription regulation by RNA polymerase II (p.adj < 0.01) (Fig. 4c, d). However, this term was also enriched in the PPI network comprising downregulated DEGs in Fig. 4a. Another PPI module comprising eight upregulated proteins showed significant enrichment for Ca^2+^-binding (p.adj < 0.0001). Interestingly, although we observed downregulated Ca^2+^-binding proteins in the PPI network of all DEGs in Fig. 3b, this term was not significantly enriched in the PPI network consisting of downregulated proteins. This suggests that genes associated with Ca^2+^-binding are uniquely enriched among the proteins upregulated under FD PSC culture conditions and may play a role in pluripotency.

These findings demonstrate that differentially expressed proteins under FD PSC culture conditions participate in shared biological networks, with specific processes uniquely enriched among downregulated or upregulated proteins, highlighting their potential significance in maintaining pluripotency.

### Ca^2+^ activity and Ca^2+^-binding proteins that are upregulated in defined PSC culture conditions are linked to the pluripotent state

Gene expression and PPI network analyses suggested that downregulated genes under the FD PSC culture conditions were associated with PSC differentiation. Therefore, we sought to explore the potential connection between genes associated with Ca^2+^-binding, uniquely enriched at the protein level among the upregulated genes in FD PSC culture conditions, and the pluripotent state. To test this hypothesis, iPSCs obtained from healthy individuals (Ctrl5^10^, Ctrl7^11,12^, Ctrl9^5^, and Ctrl14^13^) were cultured in parallel on Laminin-521 in defined E8™ and E6™ PSC media. Compared to E8™ medium, E6™ medium lacks two important growth factors, TGFβ and bFGF, which are required for maintaining pluripotency. After 7 days of culture, E6 iPSCs showed clear signs of differentiation compared with their E8 counterparts. E6 iPSCs were heterogeneous, showing either regular iPSC morphology, larger, more flattened, and/or radially organized, and more elongated morphology compared to iPSCs cultured in E8 medium (Supplementary Fig. 5A, B). Moreover, iPSCs cultured in E6 proliferated slower than those cultured in E8 medium. Upon harvesting on day 7, the total number of cells in the E6 medium was considerably lower than that in E8 medium, despite equal seeding numbers and densities (Supplementary Fig. 5C).

Since iPSCs cultured in E6 medium for 7 days displayed typical signs of differentiation, we employed flow cytometry analysis of the core pluripotency regulatory network. E6 iPSCs showed heterogeneous POU5F1 and NANOG expression, with an increased population lacking these markers compared to E8 iPSCs (Supplementary Fig. 5D). SOX2 expression was slightly increased in E6 compared to E8 culture conditions (Supplementary Fig. 5D). These results indicate that the pluripotency regulatory network was affected following 7 days of iPSC culture in E6 medium.

To investigate the association between the effects on the pluripotency network and early differentiation genes, we conducted RT-qPCR analysis to measure the expression of *AFP*, *HAND1*, *DLX5*, *NES*, and *PAX6*. *AFP* was excluded from further analysis due to low detection levels. The mesodermal markers, *HAND1* and *DLX5* were significantly and consistently upregulated in all five E6 iPSC cell lines tested (Supplementary Fig. 5E). The ectodermal marker *NES* was upregulated in E6 but did not pass multiple testing correction (Supplementary Fig. 5E). The ectodermal marker *PAX6* showed variable results, with some iPSC lines showing increased and others decreased expression in E6 media (Supplementary Fig. 5E). These findings suggest that 7 days of iPSC culture in E6 medium pushed iPSCs towards a state of unguided differentiation.

**Fig. 5:**
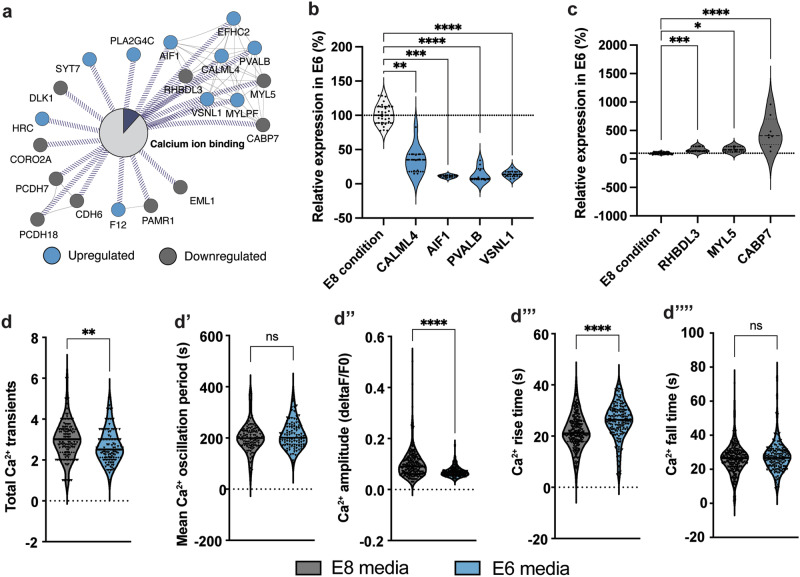
Ca^2+^ activity and Ca^2+^-binding genes that are upregulated in FD iPSC culture conditions are linked to the pluripotent state. **a** Isolated Ca^2+^-binding protein interaction network based on Fig. 3b. Blue: upregulated proteins in FD iPSC culture conditions. Gray: downregulated proteins in FD iPSC culture conditions. **b** Ca^2+^-binding genes upregulated in FD culture conditions are downregulated when iPSCs start leaving the pluripotent state. QPCR gene expression data is normalized to E8 condition (white). **c** Ca^2+^-binding genes downregulated in FD culture conditions are upregulated when iPSCs start leaving the pluripotent state. QPCR gene expression data is normalized to E8 condition (white). **d** Live Ca^2+^ imaging of iPSCs cultured in E8 media (in gray) or E6 media (in blue) in baseline conditions measuring the total number of Ca^2+^ transients (**d**), mean oscillation period (**d’**), Ca^2+^ peak amplitude (**d**”, change of fluorescence over baseline fluorescence), Ca^2+^ peak rise time (**d**”’) and Ca^2+^ peak fall time (**d**””). Pooled data of 4 different iPSC control lines plotted in violin plots, line indicate median. *p < 0.05, **p < 0.01, ***p < 0.001, ****p < 0.0001, ns not significant.

After establishing an FD culture condition mimicking UD condition with heterogenous iPSCs populations, we investigated whether Ca^2+^-binding genes from the Fig. 3b module are differentially expressed upon iPSC differentiation. A simplified representation of this module is shown in Fig. 5a. We analyzed nine interconnected Ca^2+^ ion-binding genes in this module, sharing similar protein domains. After 7 days in E6 and E8 media, expression analysis confirmed Illumina array data: genes upregulated under FD conditions (*CALML4*, *AIF1*, *PVALB*, *VSNL1*) were significantly downregulated in E6, while downregulated genes (*RHBDL3*, *MYL5*, *CABP7*) were significantly upregulated (Fig. 5b, c). These results associate upregulated Ca^2+^-binding genes in FD PSCs with pluripotency and downregulated genes with differentiation.

Since the expression and function of specific Ca^2+^-binding proteins may depend on the overall spontaneous Ca^2+^ activity in a cell, we investigated whether iPSCs cultured in E6 medium displayed changes in their baseline Ca^2+^ activity compared to iPSCs cultured in E8 medium. Upon cultivation in E6 media, iPSCs showed significant reductions in the number of spontaneous Ca^2+^ transients (*p* < 0.01), the Ca^2+^ peak amplitude (*p* < 0.0001), and a significant increase in the Ca^2+^ peak rise time (*p* < 0.0001) (Fig. 5d, d”, d”). However, other parameters, such as the mean Ca^2+^ oscillation period and Ca^2+^ peak fall time, were not significantly affected (Fig. 5d’, d””).

**Fig. 6:**
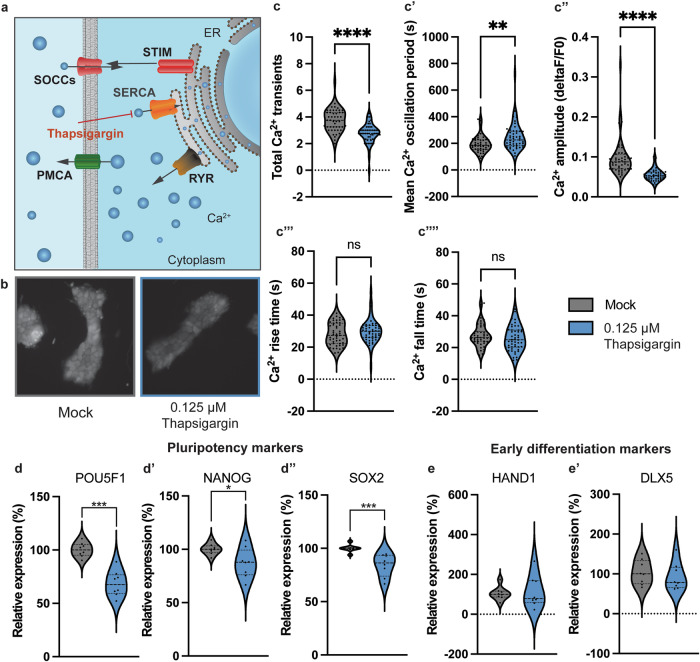
Reducing intracellular Ca^2+^ activity in FD iPSCs affects pluripotency gene expression. **a** Mechanistic display of Thapsigargin actions within the cell. **b** Morphology of FD iPSCs after 2 h of incubation with Mock or 0.125μM Thapsigargin. **c** Live Ca^2+^ imaging of iPSCs cultured in E8 media in presence (in blue) or absence of 0.125 μM Thapsigargin (in gray) measuring the total number of Ca^2+^ transients (**c**), mean oscillation period (**c**’), Ca^2+^ peak amplitude (**c**”, change of fluorescence over baseline fluorescence), Ca^2+^ peak rise time (**c**”’) and Ca^2+^ peak fall time (**c**””). **d** Expression of the genes POU5F1 (d), NANOG (**d**’) and SOX2 (**d**”) that make up the pluripotency network upon Mock (in gray) and 0.125 μM Thapsigargin (in blue) treatment. QPCR gene expression data is normalized to Mock condition (in gray). **e** Expression of the mesodermal differentiation genes HAND1 (**e**) and DLX5 (**e**’) upon Mock (in gray) and 0.125 μM Thapsigargin (in blue) treatment. QPCR gene expression data is normalized to Mock condition (in gray). Pooled data of four different iPSC control lines plotted in violin plots, line at median. *p < 0.05, ***p < 0.001, ****p < 0.0001, ns not significant.

We then investigated whether blocking intracellular Ca^2+^ activity in FD PSCs could impact the expression of pluripotency genes. By incubating FD PSCs with the SERCA pump inhibitor Thapsigargin for 2 h, we significantly reduced Ca^2+^ activity without altering the morphology of the cells (Fig. 6a–c). Notably, this 2-h Thapsigargin treatment led to a significant reduction in the expression of key pluripotency genes POU5F1, NANOG, and SOX2 (Fig. 6d). At this early time point, we did not observe changes in the expression of mesodermal differentiation markers HAND1 and DLX5 (Fig. 6e).

All this suggests that spontaneous Ca^2+^ activity is altered when iPSCs begin to differentiate and that the reduction of spontaneous Ca^2+^ activity in iPSCs directly affects pluripotency gene expression.

### Ca^2+^ activity is regulated differently in iPSCs that leave the pluripotent state

Spontaneous Ca^2+^ activity can be controlled by a tight interaction between ion channels, adhesion molecules, and receptor proteins^14^.

**Fig. 7:**
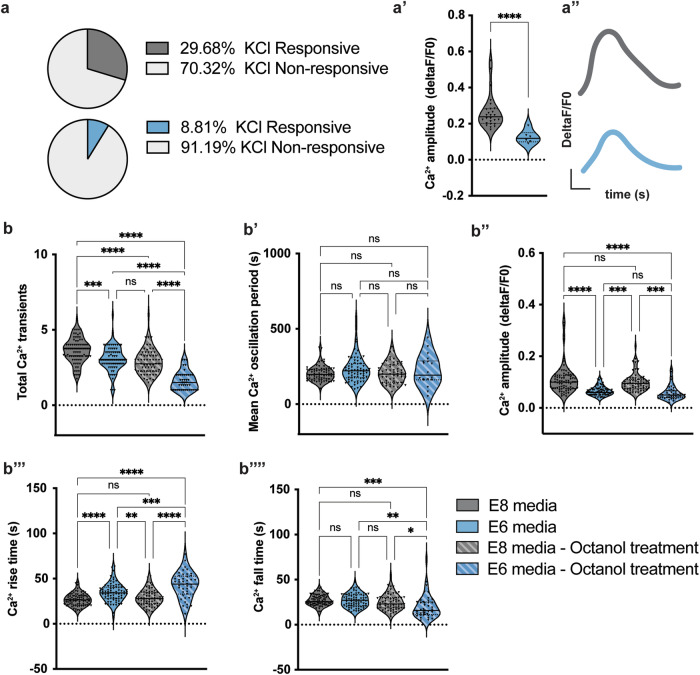
Ca^2+^ activity is regulated differently in iPSCs that leave the pluripotent state. **a** Pie chart displaying the percentage of cells cultured in E8 (in gray) and E6 (in blue) media that respond to depolarization stimuli. **a’** Ca^2+^ peak amplitude (change of fluorescence over baseline fluorescence) in response to depolarization stimuli. **a”** Representative Ca^2+^ peak of iPSCs cultured in E8 media (in gray) and E6 media (in blue) in response to depolarization stimuli. **b** Live Ca^2+^ imaging of iPSCs cultured in E8 media (in gray) or E6 media (in blue) in baseline conditions and upon octanol treatment (diagonal lines) measuring the total number of Ca^2+^ transients (**b**), mean Ca^2+^ oscillation period (**b**’), Ca^2+^ peak amplitude (**b**”, change of fluorescence over baseline fluorescence), Ca^2+^ peak rise time (**b**”’) and Ca^2+^ peak fall time (**b**””). Pooled data of 4 different iPSC control lines plotted in violin plots, line indicate median. *p < 0.05, **p < 0.01, ***p < 0.001, ****p < 0.0001, ns not significant.

To assess the impact of unguided iPSC differentiation on voltage-gated Na^2+^ (VGSCs) and Ca^2+^ ion channels (VGCCs), iPSCs cultured in E6 and E8 media were depolarized, exposing cells to a high extracellular concentration of potassium chloride (KCl). Approximately 30% of iPSCs cultured in E8 medium responded to the high KCl stimuli (Fig. 7a, in gray). In contrast, only 9% of iPSCs cultured in E6 medium responded (Fig. 7a, in blue). iPSCs cultured in E6 media that responded to KCl stimuli had a significantly reduced Ca^2+^ peak amplitude (*p* < 0.0001) compared to that of iPSCs cultured in E8 media (Fig. 7a’, a”). Although we did not specifically validate the expression levels of VGSCs and VGCCs in our E6 versus E8 iPSC culture conditions, our FD gene expression array dataset indicated a slight upregulation of VGCC T- and L-type channels (Supplementary Fig. 6). Conversely, we observed a slight downregulation in the expression of VGSCs (Supplementary Fig. 6). These results indicate that the cellular components involved in depolarization, such as VGGCs, become less important in the regulation of Ca^2+^ activity during unguided iPSC differentiation.

We next investigated the influence of cell-cell contact, such as gap junctions, on Ca^2+^ activity of iPSCs and that of differentiating iPSCs. IPSCs cultured in both E8 and E6 media displayed a significant reduction in spontaneous Ca^2+^ activity upon treatment with the gap junction blocker, octanol-1 (Fig. 7b). This effect was more pronounced for iPSCs cultured in E6 medium, which showed a significant reduction in the total number of Ca^2+^ transients (*p* < 0.0001), a significant increase in the Ca^2+^ peak rise time (*p* < 0.001), and a significant decrease in the overall Ca^2+^ peak fall time (*p* < 0.01) after octanol-1 treatment (Fig. 7b, b”’, b””). This demonstrated that cell-cell interaction is required to regulate Ca^2+^ activity at the iPSC stage and during early iPSC differentiation. However, the specific types and number of intercellular contacts influencing Ca^2+^ activity may change as iPSCs differentiate.

## Discussion

Here, we conducted a comparative analysis of Illumina array data obtained from over 100 PSC lines derived and cultured in UD and/or FD PSC culture conditions to elucidate intracellular differences and their implications for PSC characteristics. We showed that iPSCs can be generated under both FD and UD conditions. However, FD conditions largely reduces inter-line variability, irrespective of PSC type, donor sex, origin, passage number, or donor disease state.

A recent study by Buckberry et al. highlighted notable epigenetic and functional differences between iPSCs and ESCs, attributed to the preservation of gene expression and chromatin states reflective of the original somatic cells^6^. These differences, proposed to arise from Yamanaka reprogramming, may affect iPSC functionality^6^. Contrarily, Bock et al. found that some primed iPSCs align with ESCs and fall in the normal spectrum of ESC variation^7^. Given that our extensive iPSC collection is derived through Yamanaka reprogramming, we analyzed gene expression in ESCs and iPSCs under different culture conditions. Our investigation revealed culture matrices and media as primary factors influencing variation. FD culture conditions effectively minimized differences, resulting in homogenous PSC populations characterized by low levels of somatic cell marker expression. We, however, did not assess differences in the epigenetic landscape of ESCs and iPSCs cultured in UD and FD conditions.

We demonstrated that adapting UD PSCs to FD culture conditions largely reduced inter-sample heterogeneity, yielding gene expression patterns similar to PSCs originally cultivated under FD conditions. This suggests that UD PSCs can adapt their gene expression patterns and/or that FD conditions select for UD PSC populations that utilize similar pluripotency signaling pathways. Importantly, these results highlight that adapting established UD PSC cultures to FD conditions can minimize variability and enhance PSC quality.

In light of these results, we emphasize the importance of standardizing reprogramming methods and culture protocols. We recommend adopting FD culture media and matrices to enhance pluripotency robustness, consistency, and the reproducibility of iPSC and ESC differentiation protocols within and across laboratories. Further research is needed to explore how different reprogramming methods (e.g., mRNA, retroviral, episomal) and iPSC culture conditions impact large-scale variability and consistency.

The reduced variability between PSC lines under FD conditions coincided with lower germ layer differentiation marker expression and increased *NANOG* expression. Earlier studies also highlighted benefits of FD culture conditions, showing that hESCs grown on recombinant LN-521 matrices exhibited less differentiation, improved adhesion, and increased proliferation compared to Matrigel^3,15–17^. LN-521 was suggested to stabilize pluripotency gene expression and support long-term PSC proliferation and self-renewal^3,15–17^. TGFβ signaling, promoted by TGFβ1 and/or Nodal in FD media alongside bFGF, likely drives NANOG expression and strengthens pluripotency, unlike UD media using only bFGF^18^. Whether UD media or LN-521 promotes TGFβ signaling through *NANOG* remains uncertain.

*SOX2*, a marker of both pluripotency and neural progenitors^18^, was downregulated under FD PSC culture conditions in our expression dataset, potentially aligning with reduced differentiation. Indeed, upon induced differentiation, POU5F1 and NANOG levels decreased while SOX2 increased, suggesting SOX2 may act as an early differentiation indicator.

In FD PSC culture conditions, pluripotency pathways such as Hippo, Wnt, and TGFβ signaling were downregulated. This seemed to be due to reduced BMP ligand and receptor expression. No significant differences were observed downstream of TGFβ, Activin, and Nodal signaling between FD and UD conditions. The presence of TGFβ in FD media, unlike UD media, suggests that undefined components in UD media may activate multiple signaling pathways involved in pluripotency and differentiation. FD media’s simpler composition likely restricts the activation of the number of signaling pathways upstream of the POU5F1-NANOG-SOX2 network, contributing to greater homogeneity, reproducibility, and standardized differentiation potential in PSC lines.

Ca^2+^-binding proteins were significantly overrepresented among upregulated proteins under FD conditions, prompting us to investigate Ca^2+^ signaling in iPSCs. Early iPSC differentiation showed altered Ca²^+^ activity, with reduced activity frequency, lower peak amplitude, and increased rise time. High Ca^2+^ activity has been shown to be dependent on culture conditions^19^. Elevating intracellular Ca^2+^, either through GPCR agonists or SERCA pump inhibitors, can substitute bFGF in feeder-free human PSC cultures^19^. We showed that reducing intracellular Ca^2+^ activity in FD PSCs affected pluripotency gene expression. Thus, high Ca^2+^ activity is required for pluripotency.

Consistent with the observed changes in Ca^2+^ activity during early differentiation, specific Ca^2+^ binding proteins showed corresponding changes. Upregulated Ca^2+^-binding proteins in FD PSCs became downregulated during differentiation, and vice versa. This suggests a relationship between Ca^2+^ activity patterns, Ca^2+^-binding protein expression, and cell fate. Similar observations in mouse ESCs suggest Ca^2+^ activity regulates transitions between cell states^17^. Elevated intracellular Ca²^+^ inhibits naïve mESC differentiation, while chelation induces differentiation via c-Myc disruption^17^. C*-*Myc is a transcription factor involved in maintaining PSC self-renewal and pluripotency^20^. Notably, c-Myc was upregulated in FD PSCs, with its activity linked to interactions with Ca²^+^-binding proteins like calmodulin^21^.

Thus, defined culture conditions may shape specific Ca²^+^ oscillations, influencing pluripotency and differentiation through interactions between Ca²^+^ effectors and targets, such as calmodulin and c-Myc^22^. Changes in Ca²^+^ oscillations could shift effector activation, repressing pluripotency factors while activating differentiation-related genes.

High expression of Ca^2+^-binding proteins in our dataset was associated with the pluripotent state. It remains unclear whether these proteins drive pluripotency or are secondary to and regulated by Ca^2+^ activity. The upregulated Ca^2+^-binding proteins identified under FD conditions serve different functions in Ca^2+^ signaling but have not been directly linked to pluripotency. For instance, CALML4, a adhesion complex component, is essential for mechanical signal transduction^23^. Blocking cell adhesion disrupted the typical high Ca^2+^ activity displayed by FD iPSCs. AIF1 may promote cellular proliferation via MAPK signaling, which is linked to defined PSC culture conditions^24,25^. PVALB buffers Ca^2+^ ions and its expression has been linked to the maintenance of rapid Ca^2+^ dynamics^26^. Loss of PVALB was associated with increased cell body size, which was also observed during early PSC differentiation. VSNL1 is a Ca^2+^ sensing protein involved in signal transduction. It promotes cell proliferation through P2X3/P2Y2 receptor regulation, but also interacts with nicotinic acetylcholine receptors and promotes their translocation to the cell surface membrane upon depolarization stimuli^27,28^. Interestingly, we found an increased population of FD iPSCs responding to depolarization stimuli compared to those that entered differentiation, suggestion a role for VSNL1 in depolarization responses under FD conditions.

In conclusion, FD culture conditions enhanced PSC homogeneity while suppressing differentiation, potentially through regulation of Ca^2+^ signaling. Understanding these processes could facilitate FD culture standardization and advance clinical applications of human PSCs.

## Methods

### Ethical statements

This study was approved by the regional ethical review board in Stockholm, Sweden (Dnr 2016/430-31 and Dnr 2012/208-31). Written informed consent was obtained from all donors involved or from their legal guardians. All ethical regulations relevant to human research participants were followed.

### Fibroblast culture and reprogramming

Human fibroblast lines were established via mechanical dissection and enzymatic digestion (with dispase and collagenase type IA) of skin biopsies previously obtained from healthy and diagnosed individuals. Fibroblasts were cultured in IMDM (Life technologies), 10% Fetal bovine serum (Invitrogen), 1% NEAA (Gibco), and 1% Penicillin-streptomycin (Life Technologies) on 0.1% gelatin coated plates. To generate iPSCs, ~100,000 fibroblasts were transduced using non-integrative Sendai virus vectors encoding the four Yamanaka factors POUF51, SOX2, KLF4, and cMYC (CytoTune iPS Sendai Reprogramming kit, Life Technology) with a multiplicity of infection (MOI) of 3.

### PSC culture conditions

Human iPSCs and ESCs were cultured under either undefined culture conditions or defined and xenofree culture conditions as followed:

#### Undefined culture conditions

After 1 week, transduced cells were re-plated to irradiated human foreskin fibroblasts (hff, ATCC, CRL-2429) plate (1.5 × 10^6^ hff on a 100 mm 0.1% gelatin coated dish). PSC-like iPSC colonies became visible after 10 days. Colonies were picked manually using a flame-pulled pasteur pipette, and individual iPSC colony clumps were transferred to an irradiated human feeder cell coated 4-well plate 3 weeks post-transduction. To completely clear out the Sendai virus, iPSCs were split manually until passage 10. iPSC lines were cultured in standard human ES medium: Knockout DMEM (Gibco), PEST 1:100, NEAA 1:100, 20% Knockout serum replacement (Gibco) with 8 ng ng/ml bFGF (R&D). Medium was changed every day and iPSC colonies were split manually every 5–7 days. Extra colonies were frozen in a stem cell bank.

For feeder-free, undefined PSC culture conditions, iPSC lines were manually cut, and pieces were transferred to a matrigel (BD) coated plates in mTeSR Medium (Stemcell Technologies). To clear the culture from hff, iPSC lines were cultured for at least 2 more passages before samples were collected for RNA extraction.

#### Defined culture conditions

For defined feeder-free xeno-free iPSC culture conditions, Sendai virus transduced cells were re-plated on a human recombinant laminin-521 (Biolamina) coated plate (5 μg/mL) 1 week post transduction. Emerging PSC-like colonies were picked manually 3 weeks post transduction, and individual iPSC colony clumps were transferred to a fresh Laminin-521 coated plate in Essential 8™ (E8) medium (ThermoFisher Scientific). From the next and onward passages iPSC lines were split enzymatically using TrypLE Select (ThermoFisher Scientific). Single cells were plated on laminin-521 coated plates in the present of 10 μM Rock inhibitor (Y27632, Millipore). iPSC colonies were plated with the density of 15,000–20,000 cells/cm^2^, split every 4–5 days, culture medium was replaced daily, and extra cells were frozen in stem cell banks.

### Karyotyping

In brief, iPSCs at 60–70% confluency were prepared and sent for karyotyping^10,12^. The experiment was performed according to standard G-banding method using colcemid treatment to arrest cells in metaphase. Twenty to twenty-five metaphases were analyzed for each cell line using standard cytogenetic procedures.

### Illumina gene expression array

mRNA was extracted using the RNeasy kit (Qiagen) according to manufacturer’s protocol. RNA quality and quantity was determined on the Bioanalyzer (Agilent) as in ref. ^29^. RNA was amplified according to manufacturer’s protocol using the Illumina TotalPrep RNA Amplification Kit (Ambion). Next, samples were hybridized to an Illumina Gene Expression HT12 Direct Hybridization assay according to manufacturer’s protocol and run on an Illumina BeadChip reader (Illumina HT 12v.4).

### PluriTest

To characterize the pluripotency of the iPSC lines, the microarray iScan idat files were uploaded onto the online bioinformatics tool PluriTest^30^.

Pluritest compares transcriptional profiles of a sample to an extensive transcriptional profile reference set of 223 human embryonic, 41 human iPSCs, somatic cells and tissues. This analysis provides a pluripotency and novelty score. The pluripotency score indicates how strong a model-based pluripotency signature is expressed in the samples analyzed and the novelty score indicates the general model fit for a sample. All iPSCs included in this study passed both scores and were thus determined to be pluripotent (Supplementary Data 1).

### Microarray data analysis

Microarray idat files were imported into R^31^ with the miodin package version 0.5.3^32^, using probe annotation file HumanHT-12 version 4.0. The raw microarray data was first normalized with quantile normalization, and technical replicates of each biological sample were then combined by taking the mean across the technical replicates. Differential gene expression analysis was performed with limma^33^ and adjusted for sex, twin, and disease status of the samples. *P* values were adjusted for multiple testing with Benjamini-Hochberg correction. Genes with absolute log2 fold change >1 and adjusted *p* < 0.05 were considered significant. Annotation enrichment analysis of biological processes and KEGG pathways was performed with Enrichr^34^ using significant genes from the differential expression analysis as input.

### Protein-protein interaction network analysis

PPI between DEGs were obtained via the GeneMANIA plugin in Cytoscape (v.3.9.1) and included physical interactions, predicted interactions, shared protein domains, co-localization, and pathways^8^. All interactions were combined and assembled in a reference network and duplicates were removed.

The PIE algorithm was used to account for biases in the number of reported protein interactions for disease-associated genes in the generated reference PPI network^9^. PIE scores and associated *p* values were calculated against 10,000 random protein groups obtained by number-matched subsamplings selected from the reference PPI network for all DEGs, and as well separately from downregulated DEGs and upregulated DEGs.

### RNA extraction, cDNA synthesis, and quantitative PCR

To confirm the expression of DEGs in pluripotent culture conditions, iPSCs cultured for 1 week in either E6 or E8 conditions were selected for reverse transcriptase quantitative (q)PCR. Total RNA was purified from confluent 6-well plates of 5 different iPSC control lines and qPCR were performed as described with the following adaptations^35^. After cell lysis, the samples were purified on a QIAshredder column (Qiagen). Moreover, to avoid genomic DNA contamination, RNA was subjected to DNase treatment using the DNA-free kit (Ambion) before cDNA synthesis. 1 μg RNA/sample was reverse transcribed into cDNA using the iScript cDNA (Bio-Rad) according to manufacturers’ procedures.

Quantitative PCRs (qPCRs) were performed using the Fast SYBR Green Master Mix (Life Technologies) on the QuantStudio™ 5 System (Applied Biosystems). The following cycling conditions were used: initial denaturation for 20 s at 95 °C, followed by 1 s at 95 °C and 20 s at 60 °C for 40 cycles (QPCR data collection). The products were then denatured at 95 °C for 1 s and cooled to 60 °C for 20 s (melt curve data collection).

For each cell line, three biological and two technical replicates were analyzed. Differential gene expression was calculated using the 2^ΔΔCt^ method. The average Ct value for each sample was calculated and subtracted from the geometric mean Ct value of the reference genes *GAPDH* and *TBP* to calculate the ΔCt value. All primer sequences for the gene transcripts that were tested in this manuscript are provided in Supplementary Data 3.

### Flow cytometry

iPSCs grown in either E6^TM^ or E8^TM^ were washed with PBS, followed by chemical harvesting using TrypLE Select, 1.2 million cells were fixed in 250μl fixation/permeabilization buffer for 15 min at RT using the FOXP3 Transcription Factor Staining Buffer Set (eBioscience, Invitrogen). Cells were washed with permeabilization buffer and stained for 20 min in the dark with the conjugated pluripotency antibodies (1 μl/100 000 cells) listed in Supplementary Data 3. After washing, cells were resuspended in stain buffer and passed through a 35 μm cell strainer (Falcon). All samples were run on a CytoFLEX (Beckman Coulter) and analyzed with FlowJo™ v10.8 Software (BD Life Sciences).

### Calcium imaging

iPSCs grown on a LN-521 substrate in a 35 mm culture dish, were loaded with the Ca^2+^-sensitive fluorescence indicator Fluo-4/AM (5 µM; Invitrogen, USA) and Pluronic F-127 (0.625% ThermoFisher Scientific) and incubated for 20 min at 37 °C in Krebs-Ringer buffer, containing: NaCl (119 mM), KCl (2.5 mM), NaH_2_PO_4_ (1 mM), CaCl_2_ (2.5 mM), MgCl_2_ (1.3 mM), HEPES (20 mM) and D-Glucose (11 mM), with pH adjusted to 7.4, (All from Sigma). Ca^2+^-measurements were performed in Krebs-Ringer buffer at RT on an upright microscope (Carl Zeiss) equipped with a 20 × 1.0 NA lens (Carl Zeiss). Excitation was assessed at 480 nm with a wavelength switcher (DG4, Sutter Instrument) for 15 min at a sampling frequency of 0.5 Hz. The equipment was controlled with, and data was collected using MetaFluor (Molecular Devices). FIJI, MATLAB (R2021a, MathWorks, USA), and FluoroSNNAP^36^ were used to process and analyze the collected data.

### Thapsigargin treatment

FD iPSCs (Ctrl5 #II, Ctrl7 #II, Ctrl9 #I, and Ctrl14 #II) were treated with E8^TM^ supplemented with Thapsigargin (Sigma-Aldrich) at a final concentration of 0.125μM, diluted in DMSO. For the mock condition, cells received an equivalent concentration of DMSO alone. After 2 h of incubation, the iPSCs were either subjected to live Ca^2+^ imaging or harvested for RNA isolation, as described earlier. For each cell line, two biological replicates and two technical replicate per biological replicate were analyzed.

### Statistics and reproducibility

The results were plotted in R or PRISM (GraphPad Prism 9). Sample size was determined based on the amount of cell lines included in the study. Biological replicates are considered to be cell lines from different individuals. Technical replicates are considered to be either different clones from the same cell line or the inclusion of the same cell line in an experiment several times. Statistical analysis of live Ca^2+^ imaging and qPCR was done with a non-parametric Kruskal-Wallis test in combination with a Dunns multiple comparisons post-test. Non-parametric Mann-Whitney test was used to calculate differences between two conditions. *P* values lower than 0.05 were considered as significant, with *p < 0.05, **p < 0.01, ***p < 0.001, ****p < 0.0001.

### Reporting summary

Further information on research design is available in the Nature Portfolio Reporting Summary linked to this article.

## Supplementary information

Supplementary Figs.

Description of Additional Supplementary Files

Supplementary Data 1

Supplementary Data 2

Supplementary Data 3

Reporting summary

## Data Availability

All raw microarray data is available in the Swedish National Data Service under SND-ID: 2024-505 (10.48723/fr58-f782). The processed and raw data, as well as the scripts used to generate the figures, are included in the supplementary materials or are available upon reasonable request from the corresponding authors.
