# Supplementary material for: Defined culture conditions robustly maintain human stem cell pluripotency, highlighting a role for Ca2+ signaling
Source: Commun Biol. 2025 Feb 18;8:255. doi: 10.1038/s42003-025-07658-z (PMC11836331; doi:10.1038/s42003-025-07658-z)
Supplement: Supplementary file 1 — Supplementary Figs. [file 42003_2025_7658_MOESM1_ESM.pdf]

Defined culture conditions robustly maintain human stem cell pluripotency, highlighting a role for Ca^{2+} signaling.

Ilse Eidhof[1,2,*], Benjamin Ulfenborg[3], Malin Kele[1,+], Mansoureh Shahsavani[1,#], Dania Winn[1,2], Per Uhlén[2] and Anna Falk[1,4,*]

1. Department of Neuroscience, Karolinska Institutet, Stockholm, 171 11, Sweden
2. Department of Medical Biochemistry and Biophysics, Karolinska Institutet, Stockholm, 171 11, Sweden
3. School of Bioscience, University of Skövde, Skövde, 541 28, Sweden
4. Department of Experimental Medical Science, Lund University, Lund, 221 84, Sweden

Author list footnotes

[+]present address: BioLamina AB, Sundbyberg, 172 66, Sweden

[#]present address: Department of Molecular Medicine and Surgery and Center for Molecular Medicine, Karolinska Institutet, Stockholm, 171 11, Sweden

*Correspondence: anna.falk@med.lu.se, ilse.eidhof@ki.se

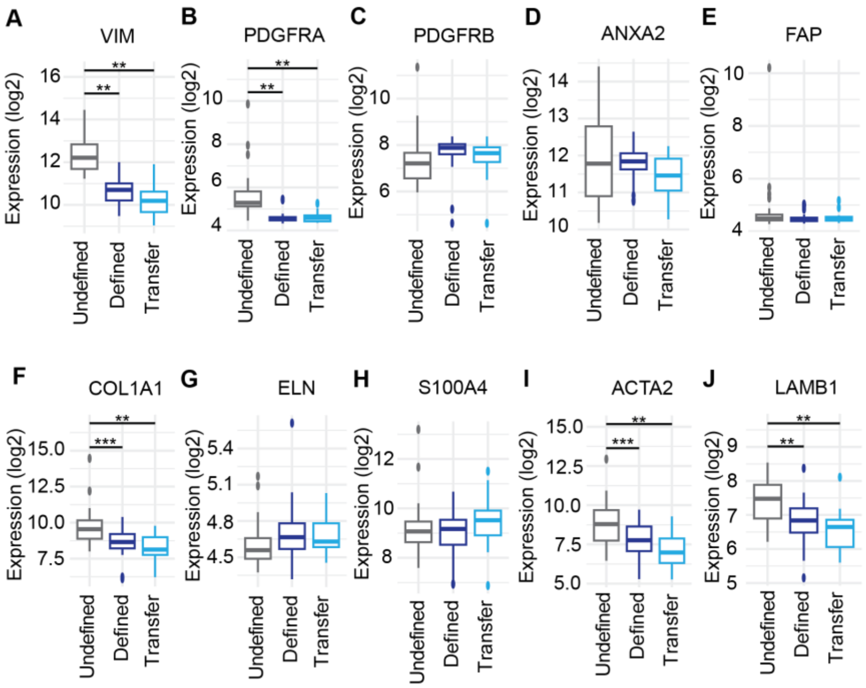

Supplementary Figure 1: A-J. Log2 microarray expression values of skin fibroblast markers for the indicated PSC culture conditions. Statical testing: one-way ANOVA followed by Tukey HSD for post-hoc testing: **p < 0.01, ***p < 0.001. Boxplots: central line is median, the box limits are the lower and upper quartiles, the whistlers represent 1.5 times the IQR and points are samples further away.

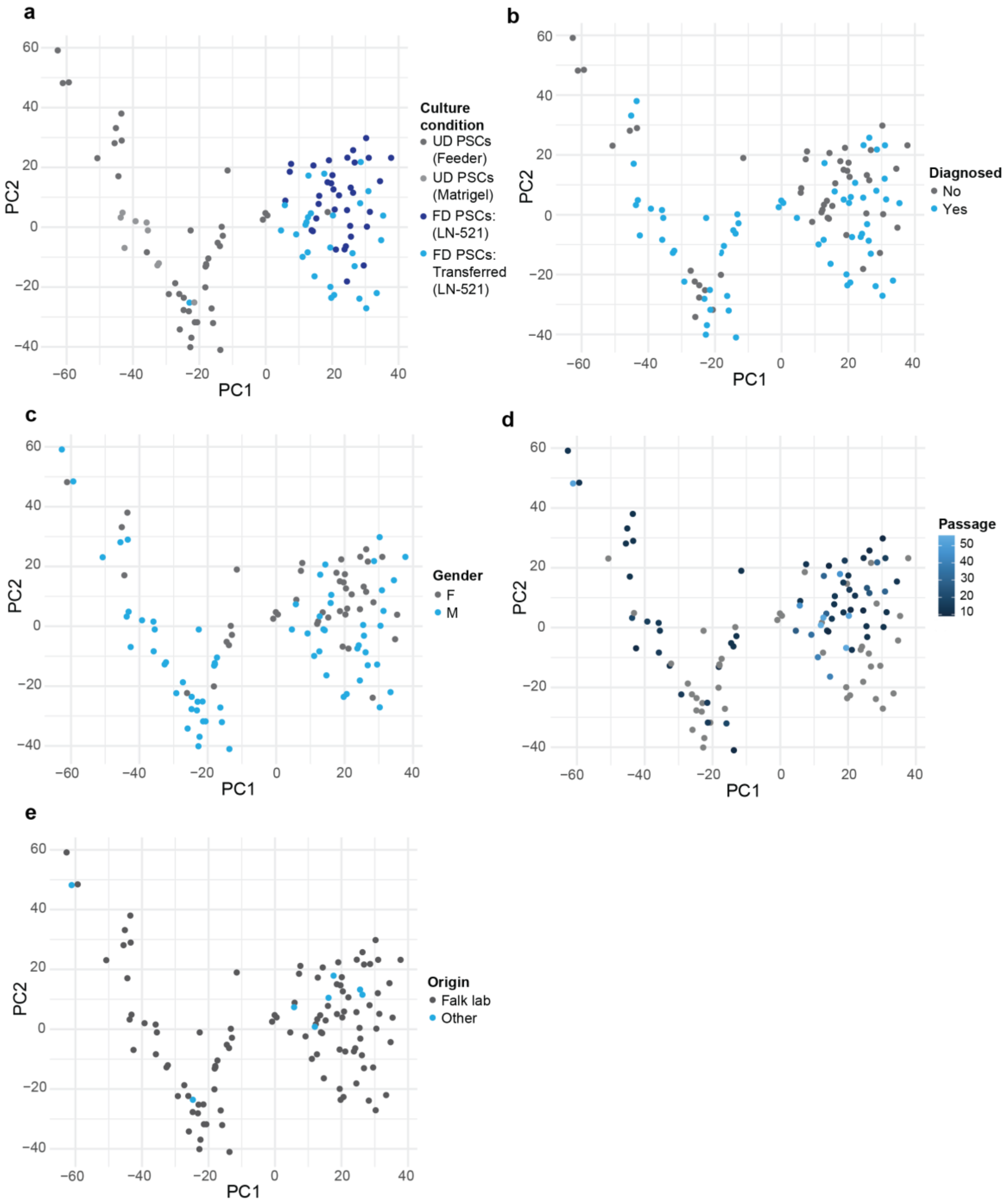

Supplementary Figure 2: PCA clustering is unaffected by potential confounding variables in the dataset. **A.** Visualization of plate coating in the PCA plot: human feeders (in dark grey), Laminin-521 (in dark blue), Matrigel (in light grey) and Transfer from feeders to Laminin-521 (in light blue). **B.** Visualization of iPSCs that originate from individuals with (in blue) or without (in grey) a diagnosis in the PCA plot. **C.** Visualization of PSCs that originate from male (in blue) or female (in grey) individuals in the PCA plot. **D.** Visualization of the passage number of the PSC line at the time of the microarray in the PCA plot. **E.** Visualization of the laboratory origin of the PSC line in the PCA plot.

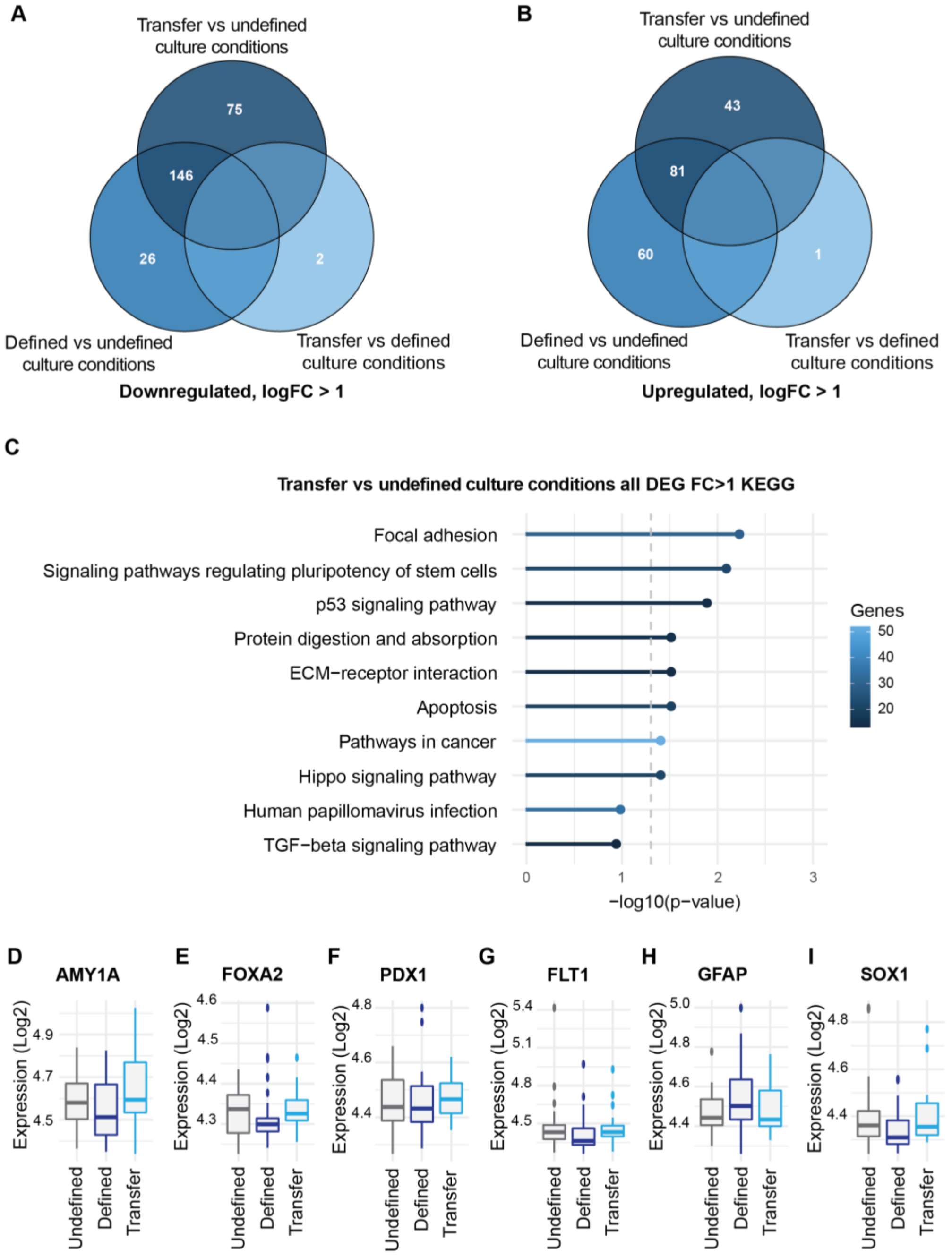

Supplementary Figure 3. A. VENN diagram showing the total number of significantly downregulated genes passing a log foldchange > 1 and between the indicated PSC culture conditions. **B.** VENN diagram showing the total number of significantly upregulated genes passing a log foldchange > 1 between the indicated PSC culture conditions. **C.** KEGG pathway analysis on DEGs between PSCs established in UD culture conditions that were transferred to FD culture conditions. **D-I.** Log2 microarray expression values of the endodermal markers *AMY1A* (**D**), *FOXA2* (**E**) and *PDX1* (F), mesodermal marker *FLT1* (**G**), and ectodermal markers *GFAP* (**H**) and *SOX1* (**I**) for the indicated PSC culture conditions. Boxplots: central line is median, the box limits are the lower and upper quartiles, the whistlers represent 1.5 times the IQR and points are samples further away.

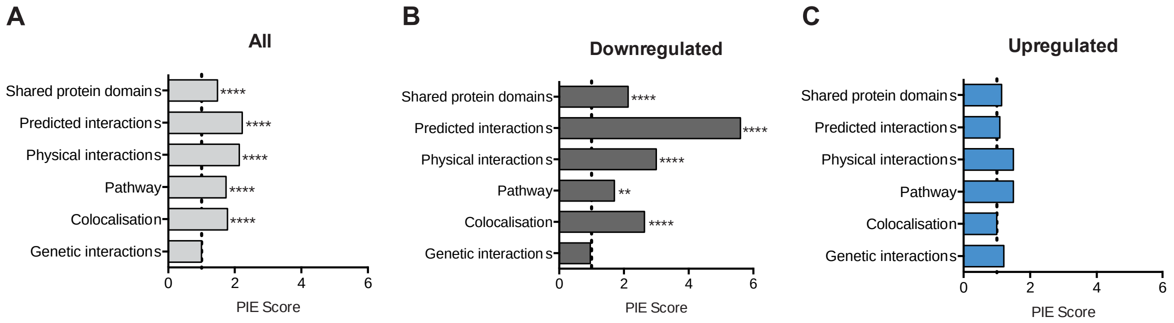

Supplementary Figure 4: Physical Interaction Enrichment Score analysis performed separately for the indicated protein-protein interaction data for all DEGs (**A**), downregulated DEGs (**B**) and upregulated DEGs (**C**) between FD and UD PSC culture conditions. **p < 0.01, ****p < 0.0001, based on 10,000 repetitions.

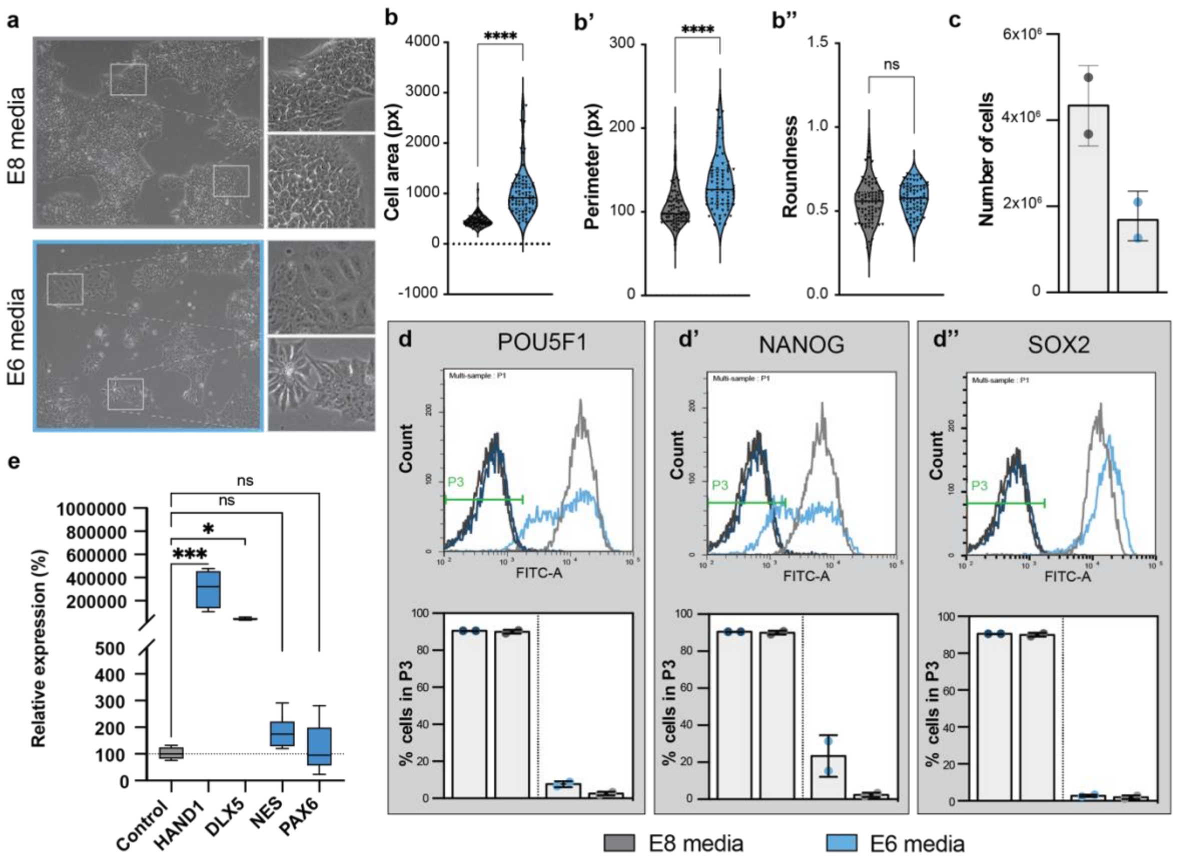

Supplementary Figure 5: Culturing iPSCs for 7 days in defined E6 culture media, without TGFβ and bFGF, induces differentiation and mimic UD culture conditions. A. Bright field images, including higher magnification zoom ins, of iPSC colonies cultured in defined E8 media (grey) and E6 media (blue), lacking TGFβ and bFGF. **B.** FIJI quantification of iPSC morphology parameters in E8 versus E6 media. B: iPSC area (in pixel), B' iPSC perimeter (in pixel). B'' Roundness. Violin plots showing all datapoints, line at median. **C.** Quantification of the number of iPSCs after 1 week in E8 (in grey) or E6 (in blue) culture media. Bargraph shows average with SD. **D.** FACS analysis of transcription factors POU5F1 (D), NANOG (D') and SOX2 (D'') that make up the core pluripotency network. Light blue: iPSCs cultured in E6 media stained for the indicated marker. The bargraphs below show the quantification of the percentage of cells that lay in the area of IgM stained cells (Green area P3 in D). Dark grey: iPSCs cultured in E8 stained with negative control: IgM. Dark blue: iPSCs cultured in E6 stained with negative control: IgM. Light grey: iPSCs cultured in E8 media stained for the indicated marker. **E.** Relative gene expression data (%) of ectodermal and mesodermal transcription factors in E6 media (data is normalized against the E8 control condition). Pooled data of 4 different iPSC control lines, Min-to-max boxplots, line at median. *$p < 0.05$, ***$p < 0.001$, ****$p < 0.0001$, ns: not significant.

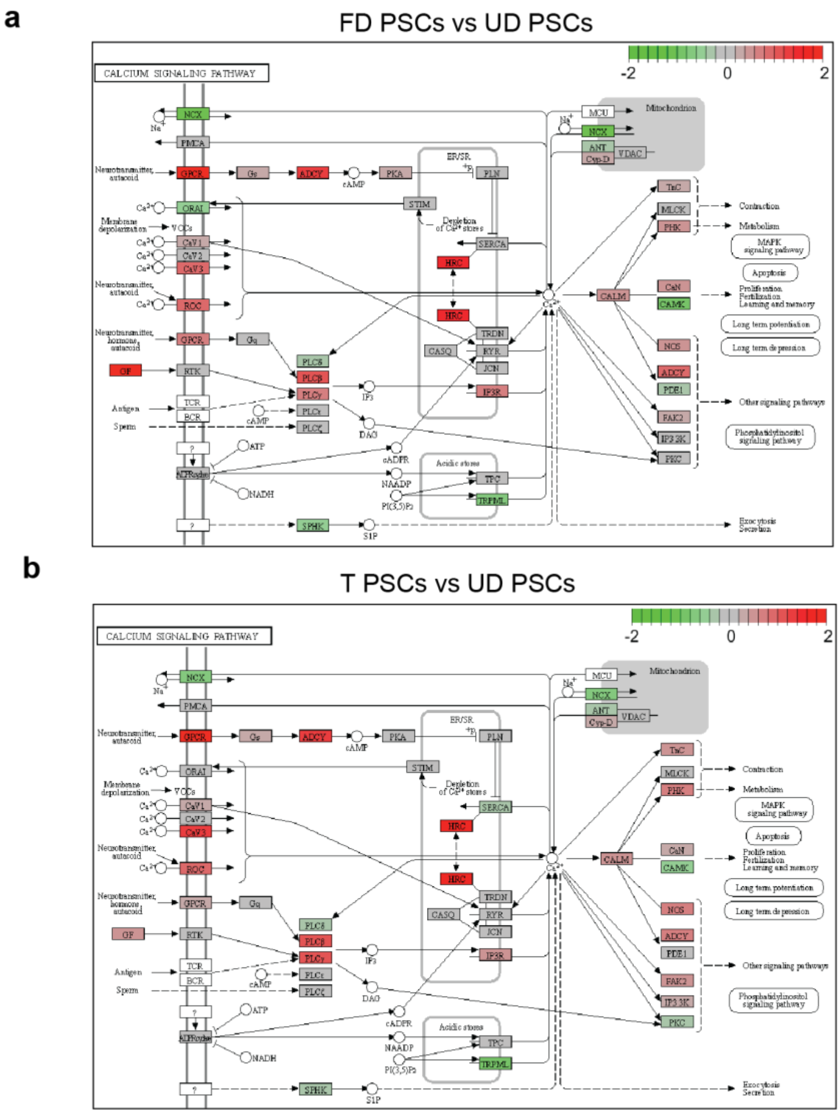

Supplementary Figure 6: Rendering of Log2 Foldchanges of genes between the indicated conditions to KEGG:hsa04020: Calcium signalling pathway using the Pathview R package. A. Log2 Foldchange of Ca^{2+} signalling gene expression between pluripotent stem cells cultured in fully defined (FD) versus undefined (UD) conditions. The data reveal an upregulation of voltage-gated calcium channel genes, as well as genes associated with Ca^{2+} signaling involving GPCRs (G-protein-coupled receptors) and the endoplasmic reticulum. Conversely, genes related to NCX (sodium-calcium exchanger) activity are downregulated under FD conditions. **B.** Log2 Foldchange of Ca^{2+} signalling gene expression between pluripotent stem cells transferred (T) to and cultured in fully defined versus undefined (UD) conditions. The observed trends in gene expression are highly similar to those in panel A, confirming the consistent upregulation of voltage-gated calcium channels, GPCR-related signaling genes, and endoplasmic reticulum calcium signaling genes, alongside the downregulation of NCX-related genes in FD conditions.
