# Supplementary material for: Defined culture conditions robustly maintain human stem cell pluripotency, highlighting a role for Ca2+ signaling
Source: Commun Biol. 2025 Feb 18;8:255. doi: 10.1038/s42003-025-07658-z (PMC11836331; doi:10.1038/s42003-025-07658-z)
Supplement: Supplementary file 2 — Description of Additional Supplementary Files [file 42003_2025_7658_MOESM2_ESM.pdf]

Description Of Additional Supplementary File

File name: Supplementary Data 1

Description: An overview of and detailed information on the PSC samples included in the analysis.

File name: Supplementary Data 2

Description: An overview of the Differential Gene Expression (DGE) analysis results utilized for generating the figures.

File name: Supplementary Data 3

Description: Key resources table.
