# Supplementary material for: Defined culture conditions robustly maintain human stem cell pluripotency, highlighting a role for Ca2+ signaling
Source: Commun Biol. 2025 Feb 18;8:255. doi: 10.1038/s42003-025-07658-z (PMC11836331; doi:10.1038/s42003-025-07658-z)
Supplement: Supplementary file 5 — Supplementary Data 3 [file 42003_2025_7658_MOESM5_ESM.pdf]

Supplementary Data 3. Key resources table.

REAGENT or RESOURCE	SOURCE	IDENTIFIER
Antibodies		
OCT3/4 (FACS)	BD Pharmigen	Cat#560217
SOX2 (FACS)	BD Pharmigen	Cat#561593
NANOG (FACS)	BD Pharmingen	Cat#560791
Rabbit monoclonal anti-Oct4A	Cell Signaling	Cat#C30A3
Rabbit monoclonal anti-Nanog	Cell Signaling	Cat#4903S
Rabbit anti-Sox2	Merck Millipore	Cat#MAB5603
Donkey anti-Rabbit IgG IRDye 680RD	LI-COR	Cat#925-68073
Goat anti-Rabbit IgG- Alexa Fluor 594	Invitrogen	Cat#A11012
Biological samples		
Skin biopsies	Ethical permits approved by Swedish ethical committee, all samples were given with informed consent from individuals participating in study.	N/A
Chemicals, peptides, and recombinant proteins		
Fluo-4, AM, cell permeant	Thermo Fisher Scientific	Cat#F14201
Pluronic™ F-127 (20% Solution in DMSO)	Thermo Fisher Scientific	Cat#P3000MP
Laminin-521	BioLamina	Cat#LN521
ROCK inhibitor (Y-27632)	Millipore	Cat#SCM075
Penicillin-streptomycin	Life Technologies	Cat#15070-063
Fetal Bovine Serum	Invitrogen	Cat#10270106
NEAA	Life Technologies	Cat#11140-076
IMDM	Life technologies	Cat#2198002-032
Knockout DMEM	Gibco	Cat#10829018
Knockout serum replacement	Gibco	Cat#10828-028
bFGF	R&D	Cat#78003.1
Matrigel	BD	Cat#356234
mTeSR medium	Stemcell Technologies	Cat#5825

Essential 8™ (E8) medium	Thermo Fisher Scientific	Cat#A1517001
Essential 6™ (E6) medium	Thermo Fisher Scientific	Cat#A1516401
TrypLE Select	Thermo Fisher Scientific	Cat#12563011
CytoTune-iPS 2.0 Sendai Reprogramming Kit	Thermo Fisher Scientific	Cat#16517
Illumina HT 12 v4 Expression BeadChip	Illumina	Cat#BD-103-0204
SuperScript III First-Strand Synthesis System	ThermoFisher Scientific	Cat#18080-051
iScript™ Advanced cDNA Synthesis Kit	BioRad	Cat#1725038
All prep DNA/RNA/Protein mini kit	Qiagen	Cat#80004
RNeasy Mini Kit	Qiagen	Cat#74106
SYBR green	ThermoFisher Scientific	Cat#4385616
Experimental models: Cell lines		
Human iPSC lines 1-17, 19-38, 42-45, 47-49	iPS Core facility Karolinska Institute	N/A
Human iPSC lines 39 and 46	Dahl laboratory Upsala University	N/A
Human ESC lines 181, 360, HS983A	Lanner laboratory Karolinska Institute	N/A
Human ESC line H9	WiCell	H9
Human fibroblast cell lines: hFib 28, hFib 103	iPS Core facility Karolinska Institute	N/A
Oligonucleotides		
GAPDH_Ex6-8 forward and reverse primer: TTCGTCATGGGTGTGAACC AGTGATGGCATGGACTGTGG	This paper	N/A
TBP_Ex6-8 forward and reverse primer: TGCTCACCCACCAACAATTTAG ACGTCGTCTTCCTGAATCCC	This paper	N/A
CALML4_Ex3-4 forward and reverse primer: CTGCGGTCAAAACTCACGAG TTGCTCCAAGTTTTCAGGCC	This paper	N/A
AIF1_Ex5-6 forward and reverse primer: TGGAGAAACTTGGAGTCCCC GGCTTTTCCTTTTCTCTCGC	This paper	N/A

PVALB_Ex4-5 forward and reverse primer:<br>AGATGGGGACGGCAAAATTG<br>TGTCATTAGAGGGCCACAGG	This paper	N/A
VSNL1_Ex2-3 forward and reverse primer:<br>TCTCGAGGAATTTCAGCAGC<br>CCTGGAGGTGATGGACAGAG	This paper	N/A
RHBDL3_Ex5-6 forward and reverse primer:<br>GTTTACCACCCACAGCTGC<br>CGTAGACAAGCCCAATTCGG	This paper	N/A
MYL5_Ex5-6 forward and reverse primer:<br>AAGGACGACGAGCTGGAC<br>GATTTTCCCTTTCCCGTCCG	This paper	N/A
CABP7_Ex2-3 forward and reverse primer:<br>CGCTCACTGGGTTACATGC<br>GTGCCATGGAACTTCTCTGG	This paper	N/A
HAND1_Ex1-2 forward and reverse primer:<br>CCAGCTACATCGCCTACCTG<br>AATCCTCTTCTCGACTGGGC	This paper	N/A
DLX5_Ex1-2 forward and reverse primer:<br>CTACGCTAGCTCCTACCACC.<br>CTTCTCTGTAATGCGGCCAG	This paper	N/A
NES_Ex2-4 forward and reverse primer:<br>ACCTCAAGATGTCCCTCAGC.<br>GAGCAAAGATCCAAGACGCC	This paper	N/A
PAX6_Ex8-9 forward and reverse primer:<br>CGGAGAAGATTCAGATGAGGC.<br>TGGCTGCTAGTCTTTCTCGG	This paper	N/A
Software and algorithms		
PluriTest	https://pluritest.org	Müller et al, Nature Methods, 2011
RStudio	https://www.rstudio.com	Version 1.1.383
R	https://www.r-project.org	Version 3.4.2
Miodin package	Ulfenborg B[32]	Version 0.5.3
Limma package	Ritchie et al.[33]	Version 2015
Enrichr	Kuleshov et al.[34]	Version 2016
FIJI	https://fiji.sc	Version 2.0.0
Cytoscape	https://cytoscape.org	Version 3.9.1

Matlab	MathWorks	Version R2021a
FluoroSNNAP	Patel et al.[34]	Version 15.04
Other		
Sequence data, analyses, and resources related to the pluritest array data	This paper	Available through the Swedish National Data Service under SND-ID: 2024-505 (DOI: 10.48723/fr58-f782) and/or on request to corresponding author.
