# Supplementary material for: Defined culture conditions robustly maintain human stem cell pluripotency, highlighting a role for Ca2+ signaling
Source: Commun Biol. 2025 Feb 18;8:255. doi: 10.1038/s42003-025-07658-z (PMC11836331; doi:10.1038/s42003-025-07658-z)
Supplement: Supplementary file 6 — Reporting summary [file 42003_2025_7658_MOESM6_ESM.pdf]

Corresponding author(s): Ilse Eidhof, Anna Falk

Last updated by author(s): Jan 20, 2025

Reporting Summary

Nature Portfolio wishes to improve the reproducibility of the work that we publish. This form provides structure for consistency and transparency in reporting. For further information on Nature Portfolio policies, see our Editorial Policies and the Editorial Policy Checklist.

Statistics

For all statistical analyses, confirm that the following items are present in the figure legend, table legend, main text, or Methods section.

n/a	Confirmed	
☐	☒	The exact sample size (*n*) for each experimental group/condition, given as a discrete number and unit of measurement
☐	☒	A statement on whether measurements were taken from distinct samples or whether the same sample was measured repeatedly
☐	☒	The statistical test(s) used AND whether they are one- or two-sided<br>*Only common tests should be described solely by name; describe more complex techniques in the Methods section.*
☒	☐	A description of all covariates tested
☐	☒	A description of any assumptions or corrections, such as tests of normality and adjustment for multiple comparisons
☐	☒	A full description of the statistical parameters including central tendency (e.g. means) or other basic estimates (e.g. regression coefficient) AND variation (e.g. standard deviation) or associated estimates of uncertainty (e.g. confidence intervals)
☐	☒	For null hypothesis testing, the test statistic (e.g. *F*, *t*, *r*) with confidence intervals, effect sizes, degrees of freedom and *P* value noted<br>*Give P values as exact values whenever suitable.*
☒	☐	For Bayesian analysis, information on the choice of priors and Markov chain Monte Carlo settings
☒	☐	For hierarchical and complex designs, identification of the appropriate level for tests and full reporting of outcomes
☒	☐	Estimates of effect sizes (e.g. Cohen's *d*, Pearson's *r*), indicating how they were calculated

Our web collection on statistics for biologists contains articles on many of the points above.

Software and code

Policy information about availability of computer code

Data collection	For Ca2+ imaging data recordings, the equipment was controlled with, and data was collected using MetaFluor (Molecular Devices). All Flow Cytometry data was collected with CytExpert software, samples were run on a CytoFLEX (Beckman Coulter). QuantStudio™ 5 software was used to collect qPCR data.
Data analysis	To calculate pluripotency and novelty scores were calculated by uploading the microarray iScan idat files onto the online bioinformatics tool PluriTest30. For analysis of gene-expression, microarray idat files were imported into R31 with the miodin package version 0.5.332, using probe annotation file HumanHT-12 version 4.0. Annotation enrichment analysis of biological processes and KEGG pathways was performed with Enrichr34. Protein-protein interactions (PPI) were obtained via the GeneMANIA plugin in Cytoscape (v.3.9.1). Flow cytometry data was analyzed with FlowJo™ v10.8 Software (BD Life Sciences). FIJI, MATLAB (R2021a, MathWorks, USA) and FluoroSNNAP36 were used to process and analyze the collected Ca2+ imaging data. The results were plotted in PRISM (GraphPad Prism 9). Statistical analysis of live Ca2+ imaging and qPCR was done with in PRISM.

For manuscripts utilizing custom algorithms or software that are central to the research but not yet described in published literature, software must be made available to editors and reviewers. We strongly encourage code deposition in a community repository (e.g. GitHub). See the Nature Portfolio guidelines for submitting code & software for further information.

Data

Policy information about availability of data

All manuscripts must include a data availability statement. This statement should provide the following information, where applicable:

- Accession codes, unique identifiers, or web links for publicly available datasets
- A description of any restrictions on data availability
- For clinical datasets or third party data, please ensure that the statement adheres to our policy

All raw expression data is deposited in the Swedish National Data Service under SND-ID: 2024-505.

Research involving human participants, their data, or biological material

Policy information about studies with human participants or human data. See also policy information about sex, gender (identity/presentation), and sexual orientation and race, ethnicity and racism.

Reporting on sex and gender	Biological sex is recorded for each cell line that was included in the study. No effects of biological sex on PSC line clustering was observed using principle component analysis (shown in Supplementary Figure 2). For further downstream analysis of Ca2 + signaling pathways, at least two male and female iPSC lines were included. No sex dependent differences were observed.
Reporting on race, ethnicity, or other socially relevant groupings	N/A
Population characteristics	Relevant co-variates for the PSC lines are provided in Table S1.
Recruitment	The donors were recruited from cohorts of patients and health controls. Donors have not been self-selected. We do not expect that the donor selection process impacted the data.
Ethics oversight	This study was approved by the regional ethical review board in Stockholm, Sweden (Dnr 2016/430-31 and dnr 2012/208-31). Written informed consent was obtained from all donors involved or from their legal guardians.

Note that full information on the approval of the study protocol must also be provided in the manuscript.

Field-specific reporting

Please select the one below that is the best fit for your research. If you are not sure, read the appropriate sections before making your selection.

☒ Life sciences ☐ Behavioural & social sciences ☐ Ecological, evolutionary & environmental sciences

For a reference copy of the document with all sections, see nature.com/documents/nr-reporting-summary-flat.pdf

Life sciences study design

All studies must disclose on these points even when the disclosure is negative.

Sample size	Sample size was determined based on the amount of cell lines included in the study. Biological replicates are considered to be cell lines from different individuals. Technical replicates are considered to be either different clones from the same cell line or the inclusion of the same cell line in an experiment several times.
Data exclusions	N/A
Replication	The experiments were performed on and could be reproduced with several different cell lines (biological replicates).
Randomization	Samples on the microarray chips were randomized to exclude potential effects of culture conditions, gender, diagnosis and passage number. For Ca2+ imaging and qPCRs, all samples/cells were included, but were not randomized. E6 and E8 condition of each cell line were imaged immediately after each other, to avoid confounding variables such as time-of-day on the imaging results.
Blinding	The samples were blinded when it comes to donors, however not when it comes to culture conditions.

Reporting for specific materials, systems and methods

We require information from authors about some types of materials, experimental systems and methods used in many studies. Here, indicate whether each material, system or method listed is relevant to your study. If you are not sure if a list item applies to your research, read the appropriate section before selecting a response.

Materials & experimental systems

n/a	Involved in the study
☐	☒ Antibodies
☐	☒ Eukaryotic cell lines
☒	☐ Palaeontology and archaeology
☒	☐ Animals and other organisms
☒	☐ Clinical data
☒	☐ Dual use research of concern
☒	☐ Plants

Methods

n/a	Involved in the study
☒	☐ ChIP-seq
☐	☒ Flow cytometry
☒	☐ MRI-based neuroimaging

Antibodies

Antibodies used: BD Pharmingen™ Alexa Fluor® 488 Mouse anti-Oct3/4
Clone 40/Oct-3 (RUO), catalog No: 560217. BD Pharmingen™ Alexa Fluor® 488 Mouse anti-Sox2
Clone O30-678 (RUO), Catalog No: 561593. BD Pharmingen™ Alexa Fluor® 488 Mouse anti-Human Nanog
Clone N31-355 (RUO), Catalog No: 560791. BD Pharmingen™ Alexa Fluor™ 488 Mouse IgG1, κ Isotype Control
Clone X40 (RUO), Catalog No: 567121.

Validation: Validation of all flow cytometry antibodies by BD Biosciences are routinely done.

Eukaryotic cell lines

Policy information about cell lines and Sex and Gender in Research

Cell line source(s): Human iPSC lines were created at the iPS Core facility at Karolinska Institute. Human ES lines were kindly provided by WiCell, the Niklas Dahl laborator at Uppsala University and Fredrik Lanner laboratory at Karolinska Institute.

Authentication: DNA (STR) profiling was done for all PSC lines using the human cell line authentication service provided by eurofins.

Mycoplasma contamination: Mycoplasma was tested for all cell lines using the GloMax Luminescence Reader (Promega) and MycoAlert® Mycoplasma Detection Kit (Lonza Cat. LTo7-318) and confirmed to be negative.

Commonly misidentified lines (See ICLAC register): N/A

Plants

Seed stocks: N/A

Novel plant genotypes: N/A

Authentication: N/A

Flow Cytometry

Plots

Confirm that:

☒ The axis labels state the marker and fluorochrome used (e.g. CD4-FITC).

☒ The axis scales are clearly visible. Include numbers along axes only for bottom left plot of group (a 'group' is an analysis of identical markers).

☒ All plots are contour plots with outliers or pseudocolor plots.

☒ A numerical value for number of cells or percentage (with statistics) is provided.

Methodology

Sample preparation: iPSCs grown in either E6TM or E8TM were washed with PBS, followed by chemical harvesting using TrypLE Select, 1.2 million cells were fixed in 250?l fixation/permeabilization buffer for 15 min at RT using the FOXP3 Transcription Factor Staining

Buffer Set (eBioscience, Invitrogen). Cells were washed with permeabilization buffer and stained for 20 min in the dark with the conjugated pluripotency antibodies (1l/100 000 cells) listed in Table S3. After washing, cells were resuspended in stain buffer and passed through a 35 um cell strainer (Falcon).

Instrument	All samples were run on a CytoFLEX (Beckman Coulter) V0-B5-R0 Flow Cytometer.
Software	CytExpert was used to collect/record the data. Flow cytometry data was analyzed with FlowJo™ v10.8 Software (BD Life Sciences).
Cell population abundance	Around 99% of the iPSCs grown in E8 media were positive for the markers POU5F1, NANOG and SOX2. This number was reduced for iPSCs grown in E6 media (as shown in Figure S5).
Gating strategy	Debris and doublets were removed from the main population of cells using FSC vs SSC gating of the negative stained sample (cells stained only with FITC). To determine the population of POUF51, SOX2 and NANOG positive cells, the P3 gate was drawn on the histogram, over the Isotype control peak. Everything in the P3 gate was considered to be negative for either POU5F1, SOX2 or NANOG.

☒ Tick this box to confirm that a figure exemplifying the gating strategy is provided in the Supplementary Information.
